# Elucidation of a dynamic interplay between a beta-2 adrenergic receptor, its agonist, and stimulatory G protein

**DOI:** 10.1073/pnas.2215916120

**Published:** 2023-02-28

**Authors:** Yanxiao Han, John R. D. Dawson, Kevin R. DeMarco, Kyle C. Rouen, Slava Bekker, Vladimir Yarov-Yarovoy, Colleen E. Clancy, Yang K. Xiang, Igor Vorobyov

**Affiliations:** ^a^Department of Physiology and Membrane Biology, University of California, Davis, CA 95616; ^b^Biophysics Graduate Group, University of California, Davis, CA 95616; ^c^Department of Science and Engineering, American River College, Sacramento, CA 95841; ^d^Department of Anesthesiology and Pain Medicine, University of California, Davis, CA 95616; ^e^Department of Pharmacology, University of California, Davis, CA 95616; ^f^VA Northern California Health Care System, Mather, CA 95655

**Keywords:** G protein-coupled receptor, G protein, norepinephrine, sympathetic nervous system, molecular dynamics

## Abstract

G protein-coupled receptors (GPCRs) represent the largest group of membrane receptors for transmembrane signal transduction. Ligand-induced activation of GPCRs triggers G protein activation followed by various signaling cascades. Understanding the structural and energetic determinants of ligand binding to GPCRs and GPCRs to G proteins is crucial to the design of pharmacological treatments targeting specific conformations of these proteins to precisely control their signaling properties. In this study, we focused on interactions of a prototypical GPCR, beta-2 adrenergic receptor (β_2_AR), with its endogenous agonist, norepinephrine (NE), and the stimulatory G protein (G_s_). Using molecular dynamics (MD) simulations, we demonstrated the stabilization of cationic NE, NE(+), binding to β_2_AR by G_s_ protein recruitment, in line with experimental observations. We also captured the partial dissociation of the ligand from β_2_AR and the conformational interconversions of G_s_ between closed and open conformations in the NE(+)–β_2_AR–G_s_ ternary complex while it is still bound to the receptor. The variation of NE(+) binding poses was found to alter G_s_ α subunit (G_s_α) conformational transitions. Our simulations showed that the interdomain movement and the stacking of G_s_α α1 and α5 helices are significant for increasing the distance between the G_s_α and β_2_AR, which may indicate a partial dissociation of G_s_α The distance increase commences when G_s_α is predominantly in an open state and can be triggered by the intracellular loop 3 (ICL3) of β_2_AR interacting with G_s_α, causing conformational changes of the α5 helix. Our results help explain molecular mechanisms of ligand and GPCR-mediated modulation of G protein activation.

## Significance

G protein-coupled receptors (GPCRs) and G proteins work together to transmit signals from various hormone and neurotransmitter molecules across cell membranes, and their activation and subsequent dissociation initiate a cascade of downstream signaling events resulting in modulation of cellular behavior. Here, we studied the interactions of a prototypical GPCR, beta-2 adrenergic receptor in its active state, with neurotransmitter norepinephrine and stimulatory G protein using multi-microsecond–long atomistic computer simulations to understand how energetic and structural changes in this system could initiate cellular signaling. Our results provided us with intrinsic molecular mechanisms, which may control G protein dissociation from GPCRs, and highlighted the importance of protein domain and ligand dynamics in this crucial biological process.

GPCRs transduce intracellular signaling via coupling to G proteins. In the heart, sympathetic nervous system (SNS) activation increases cardiac output to supply the body with oxygenated blood by raising the heart rate, the force of contraction, and conduction rate (1). SNS activation in the cardiovascular system is triggered by binding of two catecholamine neurotransmitters, norepinephrine (NE) and epinephrine (Epi), to specific cell surface adrenergic receptors (βARs in human heart), which belong to the superfamily of GPCRs (2). There are three βAR subtypes in the nonfailing human heart (75 to 80% of β_1_, 15 to 18% of β_2_, and 2 to 3% of β_3_), regulating cardiac rate and contractility by responding to NE and Epi (2, 3). Recently, β_2_AR has been the focus of therapeutic interest, partly because of its relative preservation of expression in the failing human heart (4). After binding to agonists, β_2_AR can activate the stimulatory G protein (G_s_). G_s_ is a heterotrimer consisting of an α subunit (G_s_α) and a tightly associated βγ complex (5). The G_s_α subunit harbors the guanine nucleotide-binding site and associates with the βγ complex in the inactive GDP-bound state (5). Binding of G_s_ to the agonist-bound β_2_AR results in the activation and dissociation of trimeric G proteins (5, 6). Both G_s_α and βγ can transduce a cascade of downstream signaling events which eventually regulate cardiac rate and contractility (2, 4). However, the molecular determinants and the dynamics of the ternary complex during receptor signaling transduction remain incompletely understood.

The GDP release by G protein is a preparatory step of G protein activation which takes place between two stable endpoint states: one is referred as “closed-out” with G protein closed and its βAR-interacting α5 helix outside the receptor, and the other is referred as “open-in” with G protein fully open and the α5 helix coupled to the receptor. In 2011, Rasmussen et al. crystallized the first high-resolution structure of β_2_AR-bound–G_s_ (β_2_AR-G_s_) which is a ternary complex in the “open-in” state consisting of a high-affinity agonist (BI-167107), an active-state receptor, and G_s_ (7). There G_s_α subunit adopts an open state with a largely displaced α-helical domain (G_s_αAH) and Ras-like GTPase domain (G_s_αRas) (7). More recently, a cryo-EM structure of the β_1_AR–G_s_ complex bound to another high-affinity agonist (isoproterenol) was solved, in which G_s_α subunit adopts a somewhat different but also open conformation (8). The agonist-bound structure is very distinct from the crystal structure of the receptor-free closed G_s_α–GTPγ complex (7, 9). In another work, an intermediate state of G_s_ between the GDP-bound G_s_ and GDP-free β_2_AR–G_s_ complex was proposed by Liu et al. by crystalizing an active-state structure of the β_2_AR stabilized by the last 14 residues of the G_s_α terminal α5-helix (6). Su and Zhu et al. found that β_1_AR induces a tilting of the α5 helix of G_s_α which deforms the GDP/GTP-binding pocket and accelerates GDP release (8). Goricanec et al. performed NMR spectroscopic characterization of an inhibitory Gα subunit, G_i_α1, and showed that it adopts a more open conformation in the apo and GDP-bound forms, but a more compact and rigid state in the GTP-bound form with no interaction to GPCR (5). They proposed that the apo G_i_ protein eventually binds to GTP, leading to subunit dissociation and loss of affinity to the receptor (5).

Meanwhile, there have also been multiple atomistic modeling and simulation studies of βAR conformational dynamics and transitions (10–17), their interactions with G_s_ protein (18–24) and other regulatory proteins (25–27), as well as endogenous ligand and drug binding (28–36) (recently reviewed, e.g., in refs. 37–39). Dror et al. studied the structural basis for GDP/GTP exchange in G_s_ protein coupled with or uncoupled from β_2_AR by combining long time scale molecular dynamics (MD) simulation with experimental validations (23). Alhadeff et al. explored the free-energy landscape of β_2_AR activation using coarse-grained (CG) modeling using multiple receptor and G_s_ protein conformational states (40). In a follow-up study, Bai et al. performed targeted MD simulations and free energy analysis based on the β_2_AR–G_s_α structure and found that the GDP could be released during the half opening of the binding cavity in the transition to the G_s_ open state; the potential key residues on α5 were also validated by site-directed mutagenesis (41). Enhanced sampling metadynamics simulations were used to predict energetics of small-molecule ligand binding to βARs and other GPCRs in good agreement with experimental affinities (42–45), but for the most part did not focus on the G protein dissociation and conformational transitions.

**Fig. 1.**
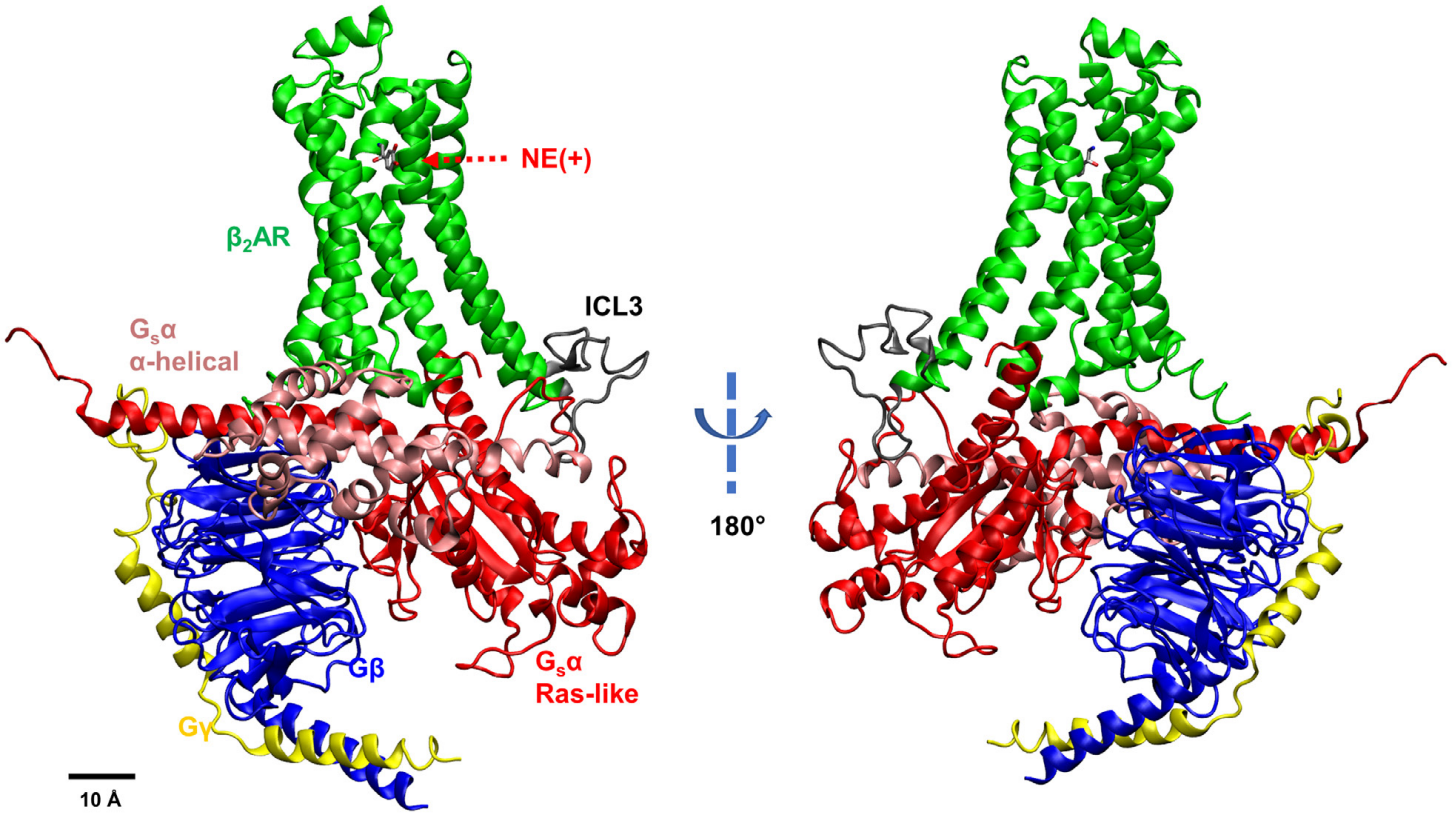
NE(+)-bound β_2_AR coupled with G_s_ protein. Different subunits and loops are illustrated by different colors (Green – β_2_AR, Gray – intracellular loop 3 or ICL3, Pink – G_s_αAH domain, Red – G_s_αRas domain, Blue – Gβ, Yellow – Gγ).

In the current study, we explore the relationship between the dissociation of G_s_ from the β_2_AR and G_s_α conformational changes, characterize the molecular determinants of how and when G_s_ may dissociate from the receptor and how the G_s_ binding affects the endogenous agonist, cationic norepinephrine, NE(+), affinity to the receptor. We performed multiple microsecond-long all-atom MD simulations to study the molecular interactions within the ternary NE(+)–β_2_AR–G_s_ complex. We applied the open-in state based on PDB:3SN6 (7) as our simulation starting point (Fig. 1) and focused on capturing the molecular conformational changes associated with the dissociation of G_s_ from the receptor.

## Results and Discussion

Two types of molecular systems were simulated: beta-2 adrenergic receptor (β_2_AR) and its complex with the stimulatory G_s_ (β_2_AR–G_s_). The cationic norepinephrine, NE(+), bound at the orthosteric binding site, was present in each system. The snapshot of the β_2_AR–G_s_ system is shown in Fig. 1. Each system was embedded in a lipid bilayer hydrated by 0.15 M NaCl, corresponding to physiological conditions in the extracellular medium and equilibrated for 90 ns using restraints that were gradually reduced in the first 40 ns of these simulations. We then performed much longer production runs. For β_2_AR, 2.5 μs Anton 2 (Anton) unrestrained MD simulations and three Gaussian-accelerated MD (GaMD) runs (600 ns each, 1,800 ns in total) were performed. For β_2_AR–G_s_ system, four different Anton runs (5.0 μs each for run 1, run 2, and run 4; 7.5 μs for run 3) and three GaMD runs (600 ns each, 1,800 ns in total) were performed (*SI Appendix*, Table S1). As we observed NE(+) partial dissociation after 4.5 μs in Anton run 3, we extended it to 7.5 μs. Based on the simulation trajectories, we first checked the dominant and secondary NE(+) binding poses in the β_2_AR and analyzed the role of G_s_ coupling in stabilizing the NE(+) binding. Then, we assessed the conformational changes in the α subunit of G_s_ (G_s_α) upon coupling with β_2_AR. The intracellular loop 3 (ICL3) of β_2_AR was found to be essential in interacting with G_s_α and causing a conformational change in the α5 helix of G_s_α. The induced α5 helix conformational change controls the formation of an active-state receptor – G protein complex. To find the molecular determinants of G_s_α conformational changes, structural parameters were analyzed, including opening/closing of G_s_α and the distance between two G_s_α domains. The geometric centers were used for all the distance and angle measurements. Finally, we analyzed distribution of those parameters converting them to two-dimensional free energy profiles to explore low-energy pathways for G_s_α conformation changes and its dissociation from β_2_AR. We also performed a posteriori implicit-solvent molecular mechanics–Poisson–Boltzmann surface area (MM–PBSA) calculations to estimate β_2_AR binding to NE and G_s_.

### Binding Affinity of NE(+) to β_2_AR and β_2_AR–G_s_.

The starting point of our β_2_AR–G_s_ simulations is the open-in G_s_α state with G_s_α in a fully open conformation and its α5 helix intruded into the intracellular part of the active-state β_2_AR (Fig. 1) which is based on the agonist-bound X-ray structure of the complex (PDB ID: 3SN6) (7). In that study, Rasmussen et al. discovered that, in the ternary complex, G_s_ binding increased the agonist-binding affinity about 100-fold compared with β_2_AR alone and that agonist binding promotes interactions of β_2_AR with GDP-bound G_s_ heterotrimer, leading to the exchange of GDP for GTP followed by the functional dissociation of G_s_ into G_s_α–GTP and βγ subunits (7). Therefore, understanding the effect of G_s_ on the agonist binding is crucial. We performed multiple microsecond-long unbiased MD simulations (Anton runs) for the NE(+)-bound β_2_AR (referred to as β_2_AR) and NE(+)-bound β_2_AR in complex with G_s_ (referred to as β_2_AR–G_s_) as shown in *SI Appendix*, Table S1. To verify some of the observations, we also performed three GaMD runs for each of the above systems (*SI Appendix*, Table S1).

**Fig. 2.**
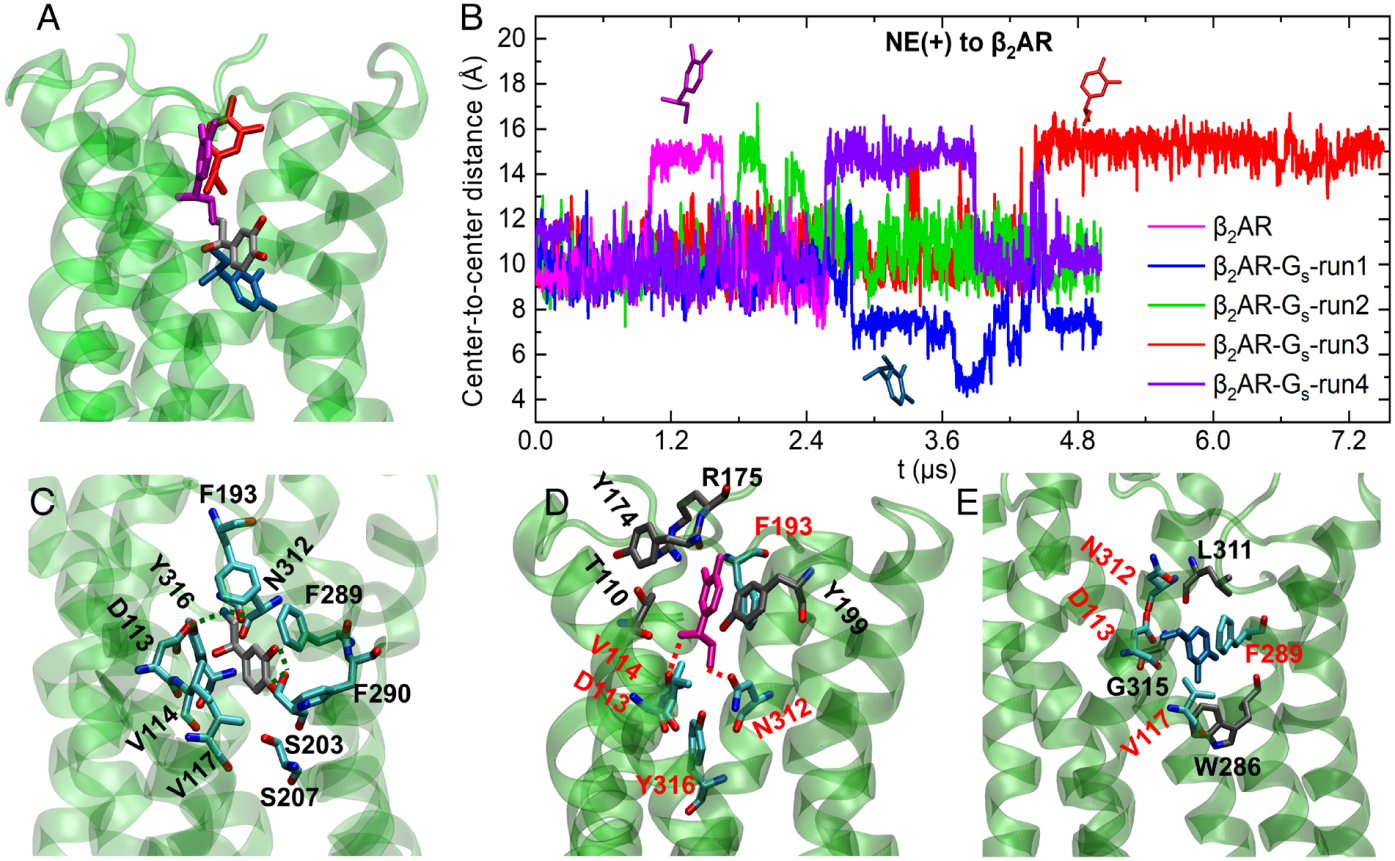
NE(+) binding poses and time series of center-to-center distances between NE(+) and β_2_AR. (*A*) The initial (gray) and three special representative binding poses of NE(+) found in β_2_AR (cluster 4 – in magenta) and β_2_AR–G_s_ (cluster 4 – in light blue and cluster 5 – in red) systems. See *SI Appendix* Fig. S1 for binding pose clustering information (*B*) Time series for center-to-center distances between NE(+) and β_2_AR (without intracellular loops) with the three special poses in panel *A* matching the plot colors. (*C*) The initial and dominant NE(+) binding pose and interacting β_2_AR residues. C atoms are shown in gray for NE(+) and in cyan for residues of β_2_AR, O atoms are in red, N atoms are in blue, H atoms are omitted. H-bonds between NE(+) and β_2_AR residues S203^5.42^, N312^7.39^, and D113^3.32^ are shown as dashed lines. (*D*) The special representative binding pose of NE(+) found in β_2_AR system cluster 4 (magenta) and interacting β_2_AR residues. H-bonds between the NE(+), N312^7.39^, and D113^3.32^ are shown as dashed lines. The preserved residues from the initial binding pocket in panel *C* are shown with cyan C atoms, whereas new residues in the binding pocket are shown with gray C atoms. (*E*) The special representative NE(+) binding pose from β_2_AR–G_s_ cluster 4 (light blue) and interacting β_2_AR residues in the binding pocket, which follow the same rendering style as in panel *D*. The geometric centers were used for the distance measurements. The Ballesteros–Weinstein (BW) numbering for the residues can be found in the text and is omitted in the figure for clarity.

We performed clustering for the NE(+) binding poses in the β_2_AR and β_2_AR–G_s_ based on their microsecond-long Anton run trajectories. Five clusters were found in each case as shown in *SI Appendix*, Fig. S1 *A*–*D*. One representative pose with the lowest root-mean-square deviation (RMSD) compared with other frames was selected for each cluster (*SI Appendix*, Fig. S1 *C* and *D*) and shown in the color-matching histogram in *SI Appendix*, Fig. S1 *A* and *B*. Fig. 2 shows the NE(+) binding results based on Anton runs. Fig. 2*A* shows the initial and three special representative poses found in the β_2_AR and in β_2_AR–G_s_ systems. The time series of center-to-center distances between NE(+) and β_2_AR for all runs are shown in Fig. 2*B* with the three special representative poses matching the colors of the plots. All other representative poses can be found in *SI Appendix*, Fig. S1 *C* and *D*. Fig. 2*C* (the gray molecule) shows the initial pose, which is also the representative pose of the biggest cluster (cluster 2 in *SI Appendix*, Fig. S1*A*) in the β_2_AR system. The amino acid residues in close contact with NE(+) forming the binding pocket were identified based on the frames collected in this cluster. The close contacts are defined as the amino acid residues within 3 Å of the NE(+) for more than half of the total MD simulation frames. The number of NE(+) poses in cluster 2 accounts for the largest proportion (28%) of the overall binding poses for β_2_AR, and it is the initial and dominant binding pose in this system [referred as NE(+)-d]. The amino acid residues forming the binding pockets of NE(+)-d are D113^3.32^, V114^3.33^, and V117^3.36^ on transmembrane helix 3 (TM3), F193^45.52^ on extracellular loop 2 (ECL2), S203^5.42^ and S207^5.46^ on TM5, F289^6.51^ and F290^6.52^ on TM6, and N312^7.39^ and Y316^7.43^ on TM7, among which D113^3.32^, S203^5.42^, and N312^7.39^ form hydrogen bonds with NE(+). The residue superscripts denote the Ballesteros–Weinstein (BW) numbering of GPCRs (46). The residues forming the binding site of NE(+) on the active β_2_AR are mainly from helices TM3, TM5, TM6, and TM7, which matches the findings of Dror et al. (12), where they observed that helices TM5, TM6, and TM7 contribute to the shift of β_2_AR conformation between inactive and active states, while the helix TM3, TM5, and TM6 interactions also play an important role in this process.

Fig. 2*D* shows the representative binding pose of NE(+) (magenta molecule) in the second biggest cluster (cluster 4) of β_2_AR [referred to as NE(+)-s1]. This binding pose is considered special because it shows a different orientation from all other poses in β_2_AR and has the biggest deviation from the initial binding pose of NE(+) in β_2_AR as shown in *SI Appendix*, Fig. S1*C*. It is also the second most abundant pose, existing in 24.7% of the simulation frames (*SI Appendix*, Fig. S1*A*). A similar NE(+) binding pose (red in Fig. 2 and *SI Appendix*, Fig. S1) is also identified in the β_2_AR–G_s_ system as cluster 5, which is also the second most abundant with 21.3% (*SI Appendix*, Fig. S1 *B* and *D*). The residues in close contact with NE(+)-s1 are identified in the same way as stated previously. Compared with the binding pocket of NE(+)-d, four new ligand-binding residues appear in the case of NE(+)-s1, which are T110^3.29^ on TM3, Y174^45.33^ and R175^45.34^ on ECL2, and Y199^5.38^ on TM5. D113^3.32^, V114^3.33^, F193^45.52^, N312^7.39^, and Y316^7.43^ are preserved in the NE(+)-s1 pocket, where D113^3.32^ and N312^7.39^ form H-bonds with NE(+), while V117^3.36^, S203^5.42^, S207^5.46^, F289^6.51^, and F290^6.52^ are not interacting with NE(+) in this pose.

Fig. 2*E* shows a special representative binding pose of NE(+) (light-blue molecule), which is captured in cluster 4 of β_2_AR–G_s_ system (*SI Appendix*, Fig. S1*D*) and is referred to as NE(+)-s2 hereafter. It shows an almost opposite orientation compared to NE(+)-s1 (Fig. 2*D*) and has an 8.85% population for the β_2_AR–G_s_ and is not represented in the β_2_AR alone (*SI Appendix*, Fig. S1). This binding pose mostly corresponds to a low-value plateau in the NE(+) to β_2_AR distance for β_2_AR–G_s_ run 1 from ~2.8 to 5 μs, as shown by a blue curve in Fig. 2*B*. Compared with NE(+)-d (Fig. 2*C*), three new interacting residues (W286^6.48^ on TM6, L311^7.38^ and G315^7.42^ on TM7) are found, while six residues (V114^3.33^, F193^45.52^, S203^5.42^, S207^5.46^, F290^6.52^, and Y316^7.43^) are missing in the binding pocket of NE(+)-s2. As noted above, the red NE(+) molecule shown in Fig. 2 *A* and *B* is another binding pose of NE(+) similar to NE(+)-s1 of β_2_AR but was found in β_2_AR–G_s_ cluster 5. It corresponds to NE(+) position plateaus in β_2_AR–G_s_ run 3 at ~3.5 μs and 4.5 to 7.5 μs (red curve in Fig. 2*B*) as well as at 2.6 to 3.9 μs of run 4 (purple curve in Fig. 2*B*).

The above results indicate that NE(+) can have different degrees of dissociation from its dominant binding pose and pocket regardless of the G_s_ binding. However, those special binding poses appear later during simulations in the β_2_AR–G_s_ cases compared to simulations with β_2_AR alone, as shown in Fig. 2*B*. The partial dissociation of NE(+) can be attributed to the β_2_AR residue movements, evidenced by the significant variations of its RMSD values, as shown in *SI Appendix*, Fig. S2*B*. We found three special representative binding poses out of 10 clusters, and only one special pose (shown in light-blue in Fig. 2) moves deeper inside the β_2_AR (based on the center-to-center distance) closer to the intracellular side. In two other special poses (shown as red and magenta in Fig. 2), we observed outward movement of NE(+) toward the extracellular side, which may indicate its partial dissociation from the receptor. Most other poses, which are dominant in both β_2_AR and β_2_AR–G_s_ simulations (Anton runs), are slight variations of the original pose with different degrees of shifting or rotation. Similar results were found in the GaMD runs as shown in *SI Appendix*, Fig. S3, where the representative binding poses were captured for both β_2_AR and β_2_AR–G_s_, except that the NE(+) in one of the β_2_AR GaMD runs almost completely dissociates from β_2_AR as shown in *SI Appendix*, Fig. S4 *A* and *B* (the gray molecule), and the full ligand dissociation may be possible to sample in longer runs and/or using ligand GaMD (LiGaMD) approach (47) to be explored in the follow-up studies.

In short, in all our MD simulations, we observed partial NE(+) dissociation, which adopted alternative binding positions in the receptor interior, in most cases closer to an extracellular side. G_s_ association in β_2_AR–G_s_ complexes seems to stabilize NE(+) binding to the orthosteric site in the β_2_AR, as was evidenced by its delayed partial dissociation (Fig. 2*B*), although a random fluctuation could potentially cause this delay. Ligand (antagonist) dissociation was also observed in an adenosine A_2A_ receptor where a multistep ligand dissociation pathway featured by different ligand poses during dissociation was suggested based on temperature-accelerated MD simulation (48). Similarly, using GaMD, different binding poses were also revealed for a partial agonist in the orthosteric pocket of a muscarinic receptor in the absence or presence of G protein mimic (nanobody) (49). These studies suggest that multiple ligand-binding poses may be common in GPCR systems with or without bound G protein.

We also computed MM–PBSA binding energies between β_2_AR and NE(+) and RMSDs for β_2_AR based on Anton runs, as shown in Table 1. In most runs of β_2_AR–G_s_, free energies of binding between β_2_AR and NE(+) are more favorable than that for β_2_AR, in agreement with the experiment (7). The reason for the stabilized NE(+) binding in the β_2_AR–G_s_ complex can be attributed to the stabilization of β_2_AR active state by the open G_s_, suggested experimentally (7) and by previous coarse-grained simulations (40). We checked the RMSDs for the β_2_AR (not including the intracellular loops) alone and in the presence of G_s_. Using the averaged β_2_AR structure as the reference, we computed the mean RMSD value and its SD for each run (Table 1) using Visual Molecular Dynamics (VMD) (50). RMSD time series for the receptor, G_s_ protein, NE(+), and the entire β_2_AR–G_s_ complex can be found in *SI Appendix*, Fig. S2. Half of the β_2_AR–G_s_ runs show lower mean RMSD values compared with the β_2_AR alone. Moreover, all the SDs (a measure of the amount of variation from the mean) for the β_2_AR–G_s_ cases are lower than that of β_2_AR alone, indicating more stable conformations of β_2_AR in complex with G_s_. These analyses confirm that NE(+) binding to β_2_AR–G_s_ is more favorable than to β_2_AR alone due to the stabilized β_2_AR structure in the complex with G_s_. In a recent GaMD study, it was also found that removal of the G protein mimic leads to a conformational transition of a muscarinic receptor M_2_ to an inactive state along with multiple orthosteric ligand dissociation and binding events consistent with extensive experimental and computational studies of other GPCRs (49).

**Table 1.** MM–PBSA interaction free energies (Δ*G*) between NE(+) and β_2_AR (in kcal/mol) along with their standard errors of mean (SEM) computed using block averages, enthalpic (Δ*H*) and entropic (–*T*Δ*S*) components, as well as mean RMSD values (in Å) along with their standard deviations (SD) for β_2_AR without loops (the average structure was taken as reference; analysis was performed for the last 2 μs of Anton trajectories)

System	Time	Δ*H*	*−TΔS*	Δ*G* ± SEM	RMSD (SD)
β_2_AR	0.5–2.5 μs	−21.61	6.88	−14.73 ± 0.92	1.65 (0.26)
β_2_AR–G_s_ – run1	3.0–5.0 μs	−27.54	11.92	−15.62 ± 2.00	1.79 (0.23)
β_2_AR–G_s_ – run2	3.0–5.0 μs	−25.09	6.10	−18.99 ± 0.44	1.56 (0.21)
β_2_AR–G_s_ – run3	5.5–7.5 μs	−23.70	7.91	−15.79 ± 0.45	1.52 (0.15)
β_2_AR–G_s_ – run4	3.0–5.0 μs	−22.42	10.81	−11.61 ± 1.11	1.67 (0.16)

See also *SI Appendix*, Fig. S14 for analysis of correlations between MM–PBSA interaction energies, β_2_AR–NE(+) distances, and RMSD values.

The MM–PBSA binding energies between β_2_AR/ β_2_AR–G_s_ and NE(+) based on GaMD runs can be found in *SI Appendix*, Table S2. Due to the nature of GaMD simulations, where different boost potentials were added to the β_2_AR and β_2_AR–G_s_ systems to accelerate dynamics of both the protein and NE(+), it is impossible to compare the binding energies between β_2_AR and β_2_AR–G_s_ systems directly, unless the energy values are reweighted properly. Despite this, it is still true that the most displaced NE(+) binds weaker to the β_2_AR or β_2_AR–G_s_, as demonstrated using nonreweighted MM–PBSA Δ*G* values for β_2_AR-GaMD run 1 as well as β_2_AR–G_s_-GaMD runs 2 and 3 (*SI Appendix*, Table S2 and Fig. S4). Since the reweighting of entropy turned out to be exceedingly noisy, we only reweighted the MM–PBSA enthalpy, Δ*H,* term by using the distribution of interaction energies based on a cumulant expansion (details can be found in the *Materials and Methods* section) as shown in the last column of *SI Appendix*, Table S2. The reweighed Δ*H* shows somewhat different trends from the nonreweighted ones, but still reflects the weaker NE(+) binding affinity in β_2_AR-GaMD run 1 and β_2_AR–G_s_-GaMD runs 2 and 3.

### G_s_ Conformational Changes after Binding with β_2_AR.

After checking the effect of G_s_ on NE(+) binding to β_2_AR, we analyzed the conformational changes of G_s_ when it couples with β_2_AR. In the published β_2_AR–G_s_ complex structure (PDB: 3SN6), used as a starting point of our simulations, the G_s_α preserves an open state with the α-helical domain (G_s_αAH) largely displaced from the Ras-like GTPase domain (G_s_αRas) as shown in Fig. 1. The G_s_αAH rotated as a rigid body with an angle of approximately 127° from the domain junction compared to the crystal structure of the closed G_s_α–GTPγ (PDB: 1AZT) (7, 9). However, a different G_s_α conformation was discovered in the complex of isoproterenol-bound β_1_AR–G_s_, which is partly based on cryo-EM, due to the dynamic nature of G_s_αAH (8). The G_s_α in β_1_AR–G_s_ is less open compared with that in the crystalized β_2_AR–G_s_ complex (7) but still can be considered as a fully open state in comparison with G_s_α alone (PDB: 1AZT) (9). G_s_α conformational transitions were thoroughly tested via long-scale MD simulations by Dror et al., who found that the separation of G_s_αRas and G_s_αAH domains occurs only in the absence of β_2_AR, whereas GDP release can only be observed after restraining G_s_α α5 in the distal conformation like that in the β_2_AR–G_s_ complex, indicating the need of an internal structural rearrangement of the G_s_αRas to weaken its nucleotide binding affinity (23).

**Fig. 3.**
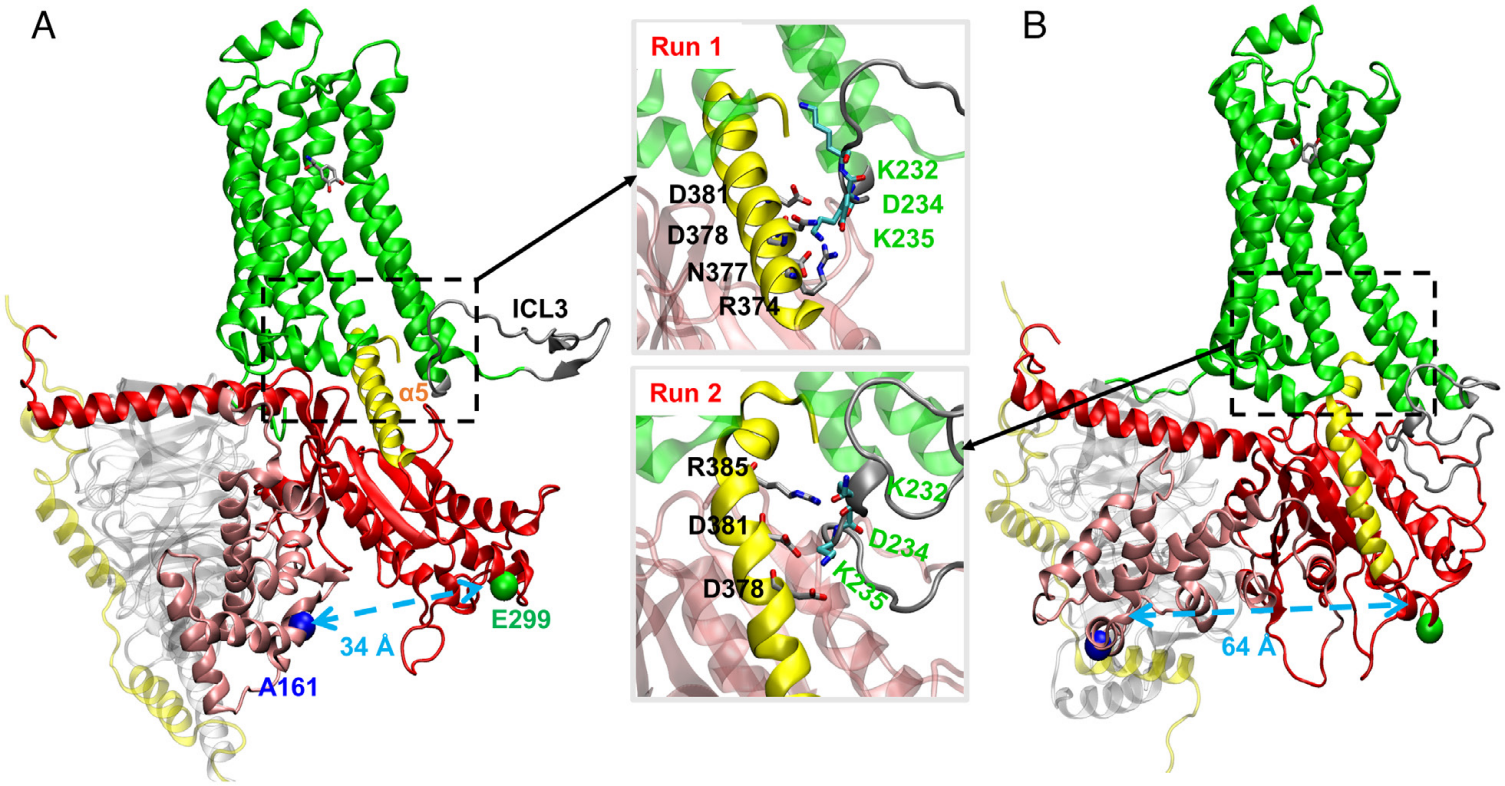
All-atom MD simulations of the active-state human β_2_AR–G_s_ with NE(+) bound based on Anton runs. (*A*) run 1 with the *Top Inset*. (*B*) run 2 with the *Bottom Inset*  Final structures are captured from the 5-μs–long unbiased MD simulation runs. Individual protein chains/subunits are labeled and shown in the ribbon representation using different colors. G_s_α α5 helix and β_2_AR intracellular loop 3 (ICL3) are colored in yellow and dark gray, respectively. C_α_ atoms of residues A161 on G_s_αAH domain and E299 on G_s_αRas domain are shown as blue and green balls, and distances between them are shown by light-blue dashed arrows. The quantification of the interactions between ICL3 and α5 helix can be found in *SI Appendix*, Table S3. The geometric centers were used for the distance measurements. The common Gα numbering (CGN) numbers (D381^G.H5.13^, D378^G.H5.10^, N377^G.H5.9^, R374^G.H5.6^, R385^G.H5.17^) for residues in G_s_α α5 as well as A161^H.HD.5^ and E299^G.HG.6^ are omitted in the figure for clarity.

As shown in Fig. 3 (based on Anton runs), we used the geometric center-to-center distance (referred to as “distance” hereafter for all the distances) between the G_s_αAH residue A161^H.HD.5^ and G_s_αRas residue E299^G.HG.6^ as an indicator for the opening and closing of G_s_α [the same one as used in the work of Dror et al. (23)], e.g., a larger distance between A161^H.HD.5^ and E299^G.HG.6^ indicates a more open G_s_α conformation. The residues are labeled by residue number and common Gα numbering (CGN) system (51) in their superscripts. The systems corresponding to different Anton simulations are referred to as runs (with GaMD runs labeled differently). If the distance is greater than or equal to 55 Å, we define G_s_α conformation as fully open; if the distance is in the range of 45 Å to 55 Å, we define it as semi-open; if the distance is in the range of 35 Å to 45 Å, then it is a semi-closed structure, and if the distance is less than or equal to 35 Å, then it is a closed structure.

Transition of G_s_α from open to closed conformation was observed, e.g., in a 5.0-μs–long MD run 1 of β_2_AR–G_s_ complex: the distance between A161^H.HD.5^ and E299^G.HG.6^ changes from 62 to 34 Å (Fig. 3*A*). Interestingly, such transition was not captured by the previous multi-microsecond–long MD simulations by Dror et al., instead, an opposite conformational change of GDP-bound G_s_α, from closed to fully open conformation, was observed but only in the receptor-free systems (23). They proposed that this conformational transition favors the closed state in the absence of the receptor (23). When it comes to the receptor-bound case, they only sampled fully open and nucleotide free G_s_α during their multi-microsecond–long MD simulations. They also proposed that the loss of GDP after G_s_ binding to β_2_AR shifts the equilibrium toward a widely open G_s_α state (23).

In run 3, we observed a very dynamic conformational transition of G_s_α between open and semi-closed states in terms of A161–E299 distance as shown in *SI Appendix*, Fig. S7*A*. This conformational transition to a semi-closed state also correlates with the increase in NE(+) to β_2_AR distance in Fig. 2*B*. Specifically, the decrease in G_s_α A161–E299 distance during ~4.0 to 5.5 μs in *SI Appendix*, Fig. S7*A* seems to correlate with an increase in NE(+) to β_2_AR distance in Fig. 2*B*, i.e., partial agonist dissociation, especially evident after ~4.5 μs. A similar, but less evident correlation can be seen for β_2_AR–G_s_ run 4, where transient rearrangements of G_s_α to a semi-closed state may be related to NE(+) partial dissociation from ~2.6 to 3.9 μs (cf. *SI Appendix*, Fig. S7*A* and Fig. 2*B*). Interestingly, G_s_α transition to a fully closed state in β_2_AR–G_s_ run 1 discussed above may eventually lead to a decreased NE(+) to β_2_AR distance at ~2.8 μs, i.e., agonist movement deeper toward the intracellular side (Fig. 2*B*). These trends indicate the potential correlation between NE(+) binding poses and G_s_ conformational changes.

In another β_2_AR–G_s_ simulation run (run 2), we observed similar open G_s_α conformation as was observed in Dror et al.’s work (23) throughout the entire 5 μs-long MD simulation (Fig. 3*B* and *SI Appendix*, Fig. S7*A*). Interestingly, in that run, we observed partial unwinding of the G_s_α α5 helix (referred to as α5), a key interaction site with the receptor (Fig. 3 *B*, *B**ottom Inset*). We correlate this α5 conformational transition with the interaction between G_s_α and flexible ICL3 of the β_2_AR as will be discussed below. Snapshots for other β_2_AR–G_s_ runs can be found in *SI Appendix*, Figs. S5 and S6, where different levels of G_s_α closing and opening, different G_s_α conformations, and interaction details between α5 and ICL3 are shown.

Due to its unstructured nature, ICL3 region is either unresolved or completely removed and replaced by T4-lysozyme (T4L) in experimental structures (15). Thus, very limited experimental (52) and simulation (15) studies have discussed the possible effect of ICL3 on the intrinsic dynamics of the receptor. Ozcan et al. found through MD simulation that ICL3 contributes to a transition of β_2_AR to a “very inactive” conformation (15). DeGraff et al. explored the function of ICL3 of α_2_-adrenergic receptors in determining subtype specificity of arrestin interaction (52). Yet, it is well accepted that direct interaction of ICL3 with G-proteins probably has a significant role in the receptor’s dynamics and the activation/inactivation pathways (12, 15). However, due to the absence of ICL3 in receptor structures, its function is not well understood. We examined specific interactions between ICL3 and G_s_α α5 as shown in the *Insets* of Fig. 3 and *SI Appendix*, Fig. S5, where the key interacting amino acid residues are labeled. K232, D234, and K235 are the common amino acid residues from ICL3 involved in the interactions with α5 in both run 1 and run 2. *SI Appendix*, Table S3 shows the number of amino acid residues in close contact between different parts of the proteins. The amino acid residues in ICL3 run 2 interact more extensively with α5 with 72.5% average percentage interaction time compared to those in run 1 with 65.7% average percentage interaction time. With the partial unwinding of α5 in run 2, the number of amino acid residues in the entire β_2_AR in close contact with α5 is reduced to 22 with 85.0% average percentage interaction time compared to 26 amino acid residues with 86.7% average percentage interaction time in run 1, indicating partial dissociation of α5 from the β_2_AR interior in run 2. These analyses suggest that ICL3 involvement may trigger the conformational change of G_s_α α5, which favors the dissociation of α5 from the β_2_AR interior. Moreover, the conformational change of α5 is not correlated with the opening and closing of G_s_α, because we observed no significant changes in α5 conformation with closed G_s_α in run 1 (Fig. 3*A*), with partially open G_s_α in runs 3 and 4 as shown in *SI Appendix*, Fig. S5, and with open G_s_α in the GaMD simulations (*SI Appendix*, Fig. S6). An important question arises here: Is there any correlation between different protein domains and what is the relationship between the G_s_ conformational changes and its dissociation?

**Fig. 4.**
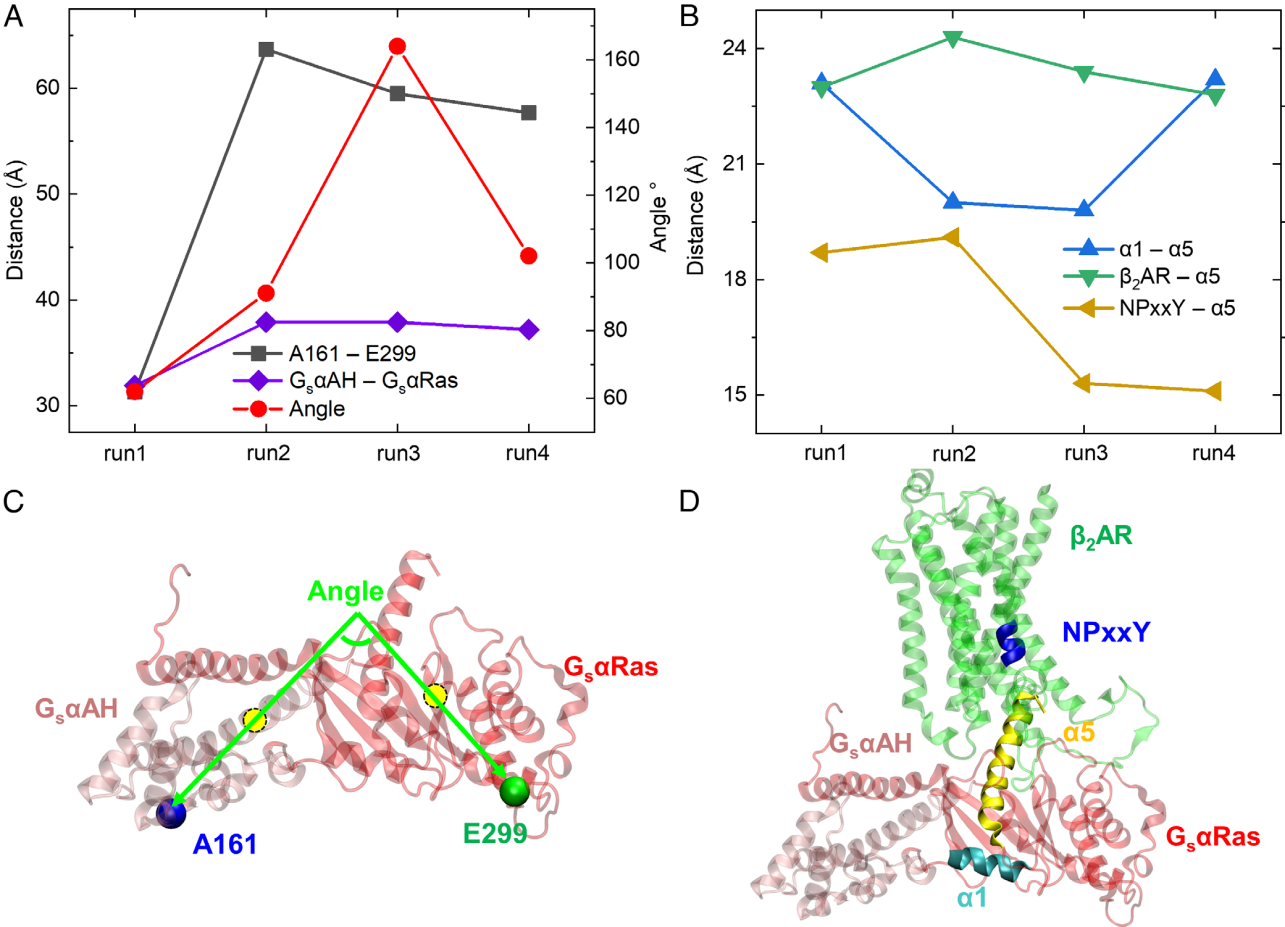
Analysis of G_s_α conformation and its possible partial dissociation from β_2_AR based on all-atom MD Anton runs. The distances and angle shown in each run are based on their average values during the last 2 μs of MD simulations. The distances and angles were measured between geometric centers of protein residues or domains. (*A*) A161–E299 distances indicating G_s_ protein conformational change (opening or closing), G_s_αAH–G_s_αRas distances indicating relative movement between the two domains, the angle between the two vectors of G_s_αAH and G_s_αRas domains indicating their relative orientation (*B*) α1–α5 distances indicating relative movement between α1 and α5 helices in G_s_α, β_2_AR–α5 distances indicating possible partial dissociation of G_s_α α5 helix from the receptor, and β_2_AR NPxxY motif–α5 helix distances also indicating G_s_α α5 partial dissociation. (*C*) Illustration of the angle between G_s_αAH and G_s_αRas domains; vector 1 goes through G_s_αAH and A161 centers; vector 2 goes through G_s_αRas and E299 centers. (*D*) Illustrations of G_s_α α5 helix (yellow), α1 helix (cyan), and β_2_AR NPxxY motif (blue helix on transmembrane domain 7).

To answer this question, we performed analysis of time series for multiple distances and angles between different protein residues and domains based on Anton runs as shown in *SI Appendix*, Fig. S7. The average values of those distances and angles based on the last 2 μs simulation for each run are shown as scatter plots in Fig. 4 *A* and *B*. *SI Appendix*, Fig. S7*A* shows the time series of A161–E299 distance. A special attention should be given to run 3, where the distance between A161 and E299 (51 Å at the end of the run) indicates a partially open structure, but it represents a closed G_s_α as shown in *SI Appendix*, Fig. S5*A*, because the G_s_αAH domain flipped upward with A161 pointing up. We then analyzed an angle between two vectors representing GsαAH and GsαRas domains indicating their relative orientation (*SI Appendix*, Fig. S7*B*). As shown in Fig. 4*C*, vector 1 goes through the centers of the G_s_αAH domain and residue A161 and vector 2 goes through the centers of the G_s_αRas domain and residue E299. Time series of G_s_αAH–G_s_αRas center-to-center distance, NPxxY–α5 distance, β_2_AR–α5 distance, and α1–α5 distance are shown in *SI Appendix*, Fig. S7 *C*–*F*.

As demonstrated using different distance and angle measurements in Fig. 4 and *SI Appendix*, Fig. S7, we captured different conformations of G_s_α in our multiple microsecond-long Anton simulations for β_2_AR–G_s_. The closing/opening conformational transition of G_s_α is due to the movement of G_s_αAH relative to G_s_αRas. G_s_αAH moves more like a rigid body as shown in RMSD plots when this domain is aligned with β_2_AR or itself (*SI Appendix*, Fig. S8), which is in line with experimental findings (7, 53). The initial distance between A161 and E299 is about 62 Å based on the crystal structure PDB: 3SN6. In run 1 (*SI Appendix*, Fig. S7*A*), we mostly captured the closed G_s_α, resembling the closed inactive G_s_α (PDB: 1AZT) (9), with the final distance of ~34 Å, as shown in Fig. 3*A*. In run 2, G_s_α goes through a short period of partial closing with a minimum distance of ~47 Å at the very beginning of the run, but the dominant conformation is fully open with a distance of ~64 Å (*SI Appendix*, Fig. S7*A* and Fig. 3*B*). In both run 3 and run 4, G_s_α shows dynamical nature, switching between fully open and semi-open states (*SI Appendix*, Fig. S7*A*). The GαAH flexibility is a reason for its low electron density in the recent cryo-EM structure of the β_1_AR–G_s_ complex (8, 53). As mentioned in the previous section, run 3 shows the flip-up G_s_αAH orientation, but it cannot be identified by A161 to E299 distance. Thus, we analyzed the angle between G_s_αAH and G_s_αRas domains and the distance between the G_s_αAH and G_s_αRas centers (*SI Appendix*, Fig. S7 *B* and *C*). The angle is defined by two vectors shown in Fig. 4*C*. This angle weakly correlates with the opening and closing of G_s_α (Fig. 4*A*); specifically, the big separation of A161 and E299 in run 2 does not guarantee a large interdomain angle, indicating seemingly random drifting of the domains in 3D space during conformational change of G_s_α. The Pearson’s correlation coefficients (*SI Appendix*, Table S4), *r*, were calculated among the data points in Fig. 4 *A* and *B* collected from the average values of the last 2 μs of each Anton runs. The value of *r* for the interdomain angle and A161–E299 distance is 0.61, validating a relatively weak correlation.

To track a possible partial dissociation of G_s_ from β_2_AR, we analyzed the distance between G_s_α helix α5 and the conserved motif NPxxY in β_2_AR’s transmembrane domain 7 (TM7) (*SI Appendix*, Fig. S7*D*) as done by Miao et al. in their GaMD simulations of adenosine receptors, a different group of GPCRs, (54). Our β_2_AR–G_s_ Anton runs 1 and 2 show almost identical displacement of α5 with the largest dissociation distance among all the runs, but this does not match with our previous analysis of dissociation in terms of the number of amino acid residue contacts (*SI Appendix*, Table S3), where run 2 shows a more dissociated β_2_AR–G_s_ complex than that of run 1. Thus, we think that the NPxxY to α5 distance may be not suitable to accurately predict displacement of α5 from β_2_AR in our systems, because NPxxY motif can be easily affected by the relative movement of TM7 to other TMs in our systems, which adds random noise into the measured distances. As α5 is a major element of the G protein–GPCR-interacting interface (8, 23, 41, 54), researchers in a recent study used it as a cognate peptide to probe the kinetics of its binding to and activation of β_2_AR, which is at least on the order of seconds (55), much longer than a time scale of our MD simulations. Despite this, we think that the center-to-center distance between β_2_AR and α5 may be suitable to check the displacement of α5 from β_2_AR which can be used as a sign for a commencement of G_s_ dissociation, and the corresponding plot is shown in *SI Appendix*, Fig. S7*E*. However, there is still no obvious correlation between the G_s_α conformational change and β_2_AR–G_s_ partial dissociation as the values of *r* between β_2_AR–α5 distance and A161–E299 distance is 0.53, G_s_α interdomain orientation angle is 0.07, and G_s_αAH–G_s_αRas distance is 0.46 (*SI Appendix*, Table S4, row 4). These results indicate that closing or opening of G_s_α by itself cannot control the suggested partial dissociation of G_s_ from β_2_AR. Instead, the internal arrangement of protein secondary structure elements may matter. To validate our assumption, we further analyzed the center-to-center distance between G_s_α helices α1 and α5 as shown in *SI Appendix*, Fig. S7*F* (the illustration of these two helices in G_s_α is shown in Fig. 4*D*). We found a strong negative correlation between α1–α5 distance and β_2_AR–α5 distance with the *r* of –0.80. The temporal variation of value of *r* between α1–α5 distance and β_2_AR–α5 distance in each Anton run was also calculated in terms of lag time (*SI Appendix*, Fig. S9). The negative correlation was found in runs 2, 3, and 4 when the lag time is less than 1 μs and where conformational transition is clearly seen in the latter two runs. Thus, we think that the stacking of α1 and α5 mostly causes the dislocation of α5 from β_2_AR. Importantly, we also found that the opening of G_s_α (indicated by G_s_αAH–G_s_αRas interdomain distance and A161 to E299 distance) is negatively correlated with the α1–α5 distance with relatively large *r* values of –0.65 (*SI Appendix*, Table S4, row 5). This indicates that the opening of G_s_α in the nucleotide free state is related to the stacking of α1 and α5 following the dislocation of α5 from β_2_AR. However, the direct correlation between G_s_αAH–G_s_αRas interdomain distance and β_2_AR–α5 distance with an *r* of 0.46 is not as strong as expected, indicating the importance of the internal domain rearrangement in the suggested partial dissociation of G_s_. The role of α1 and α5 movements has been highlighted in the structural analysis of β_2_AR–G_s_ coupling/association and GDP release processes (56). Specifically, it was found that α5 interacts with α1, β2, and β3 through highly conserved hydrophobic contacts in the GDP-bound closed G_s_α, and the structural perturbation of α1 accelerates GDP release and opening of inactive G_s_α (56). Here, in our study of G_s_ partial dissociation, α1 and α5 were found to be important in regulating the conformational change of G_s_α. The stacking of α1 and α5 may cause the opening of G_s_α (or vice versa), pulling the α5 away from the interior part of β_2_AR, which facilitates the G_s_ dissociation. In the GaMD runs, the G_s_α is almost always in a fully open state (*SI Appendix*, Figs. S6 and S10), except at the end of β_2_AR–G_s_-GaMD-run2 where a semi-open state appears. We did not see large G_s_α conformational changes in the enhanced sampling GaMD runs as observed in the unbiased Anton runs 1 and 4 which could be due to random fluctuations. We do not anticipate any correlations for the interdomain distances when there is no obvious G_s_α conformational change. In our study, we used general GaMD methodology, which boosts the overall potential of the system (57) and may not have been sufficient to trigger a G_s_α conformational transition. Using a more directed approach such as protein–protein interaction-GaMD (PPI-GaMD) (58) may solve this issue in the follow-up studies.

**Fig. 5.**
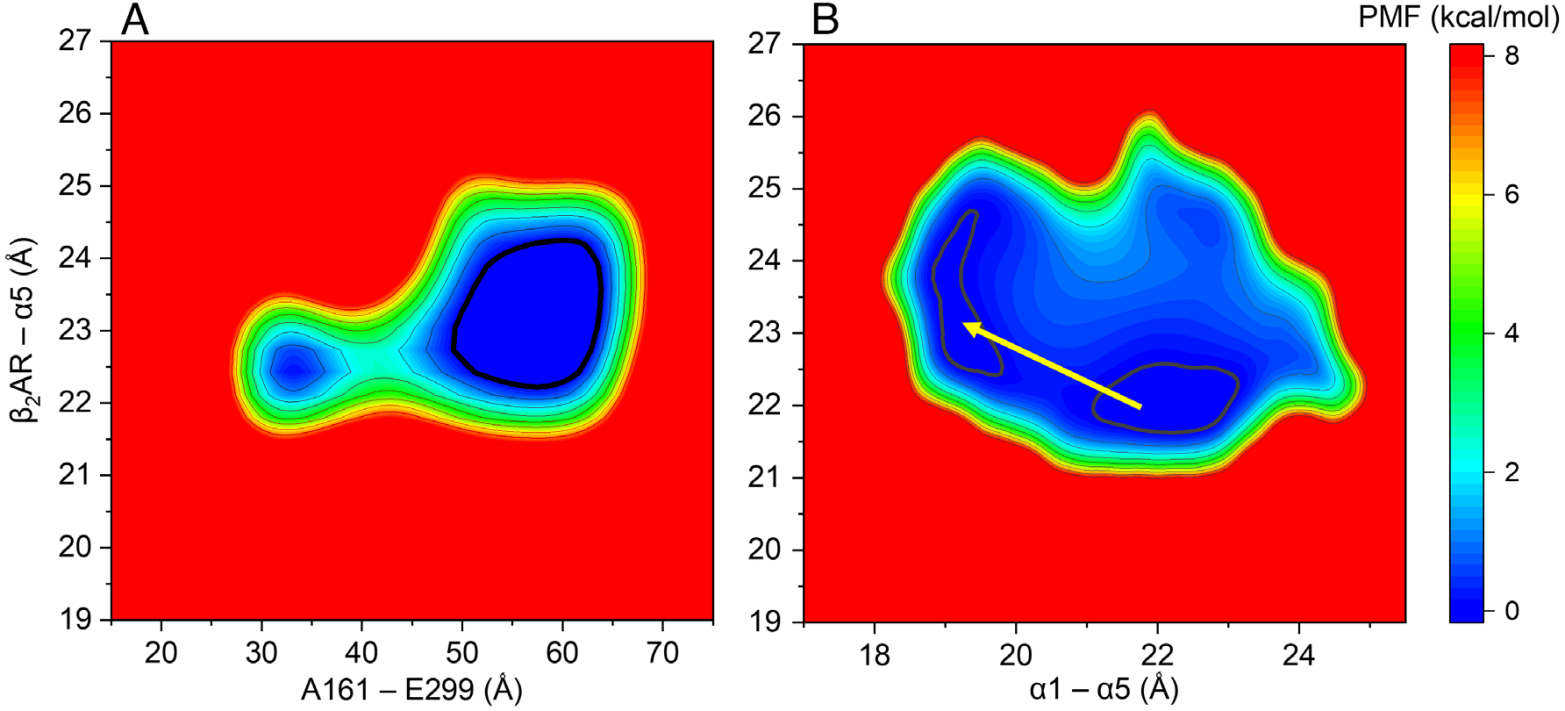
2D potential of mean force (PMF) or free energy profiles (in kcal/mol) based on G_s_α conformation and its possible partial dissociation from β_2_AR based on all-atom Anton MD simulations of the active state of the human β_2_AR–G_s_ complexes with NE(+). The 0.5 kcal/mol contour lines are shown as bold black curves. Relative free energy values from 0 to 8 kcal/mol are indicated by different colors from blue to red. All distances were measured between geometric centers of protein residues or domains. (*A*) A161–E299 distance indicating G_s_α opening or closing is shown as *X*-axis; distance between G_s_α α5 and β_2_AR indicating possible partial G_s_ dissociation is shown as *Y*-axis. (*B*) G_s_α α1–α5 distance is shown as *X*-axis; distance between G_s_α α5 and β_2_AR is shown as *Y*-axis. The contour lines are smoothed for better visualization.

We then calculated the free energy or potential of mean force (PMF, in kcal/mol) 2D profiles (Fig. 5 and *SI Appendix*, Figs. S11 and S12) based on G_s_α conformation and its β_2_AR partial dissociation to further validate the correlation analyzed in the previous section. As shown in Fig. 5*A*, the 2D PMF for the A161–E299 distance on the *x*-axis versus the β_2_AR–α5 distance on the *y*-axis exhibits two free energy minima, the closed G_s_α (at *x* = ~32 Å) and the open G_s_α (at *x* = ~58 Å). There is a small free energy barrier of about 2 to 3 kcal/mol between the two minima, but the open state is more energetically favorable, which is in line with the proposition in the earlier work of Dror et al. (23). Interestingly, only one minimum was found in the GaMD run (*SI Appendix*, Fig. S11*A*) at an even more open G_s_α state (*x* = ~67 Å). It can also be seen that the open G_s_α (Fig. 5*A*) favors a larger distance between α5 and β_2_AR compared with the closed G_s_α. Notably, there are also more chances for the dislocation of α5 from its β_2_AR binding site when G_s_α is open because of the bigger area within the 0.5 kcal/mol low-energy contour line associated with the open state. Similarly, *SI Appendix*, Fig. S12*E* shows the 2D PMF for the G_s_αAH–G_s_αRas interdomain distance versus the β_2_AR–α5 distance, also indicating a larger chance of α5 dislocation in the open state. However, the open G_s_α conformation by itself cannot guarantee the dissociation, as the structures in runs 3 and 4 at around 3 μs (*SI Appendix*, Fig. S7*A*) correspond to the open G_s_α, but they are not in a suggested partially dissociated state (*SI Appendix*, Fig. S7*E*). We previously proposed that some internal structural rearrangements may occur during the opening and closing of G_s_α, triggering the dissociation. We again found that the relative movement between G_s_α helices α5 and α1 is well correlated with the dislocation of α5 from β_2_AR. As shown in Fig. 5*B*, decreasing the distance between G_s_α α5 and α1, as marked with the yellow arrow, can lead to the dislocation of α5 with minimal energy barriers (~0.1 kcal/mol). Also, *SI Appendix*, Fig. S12*B* shows the 2D PMF for the G_s_αAH–G_s_αRas interdomain distance versus G_s_α α1–α5 interhelical distance, which exhibits a negative correlation in line with the Pearson’s correlation coefficient calculations in the previous section. These analyses indicate that the stacking of α1 and α5 helices can be the molecular determinant for the partial dissociation of G_s_ from β_2_AR in the absence of guanine nucleotide binding. The interaction between α1 and α5 was previously found to be important in the allosteric activation of G_s_α using structural and phylogenetic analyses (51). The interruption of the contacts between α1 and α5 was found to be the key step for GDP release during the association of G_s_α to its receptor (51). And, in our study, we observed that the interaction between α1 and α5 favors suggested partial dissociation of G_s_α from its receptor, thus sharing similar structural rearrangements to their association process. This indicates that interaction between α1 and α5 could be a molecular control for the association and dissociation kinetics of G_s_α and β_2_AR.

To estimate the relative binding affinities between the G_s_ and β_2_AR, we calculated corresponding MM–PBSA interaction energies as shown in Table 2. These results can be compared with different conformations of G_s_α (Fig. 3 and *SI Appendix*, Fig. S5) to give insights into the correlation between G_s_ conformation and its possible partial dissociation from β_2_AR. As discussed previously, during the last 2 μs, run 1 corresponds to the fully closed G_s_α; run 2 has a fully open G_s_α; and in run 3 and run 4, G_s_α is very dynamic, transitioning between open and intermediate states, which makes predicting the trends in MM–PBSA interaction energy challenging. Run 1 with the final closed G_s_ conformation shows the lowest (most favorable) free energies of binding, while run 2 with a fully open structure shows relatively higher (less favorable) binding free energy, indicating more chances of G_s_ dissociation with the open state. This result is in line with the 2D PMF analysis (discussed above) where the minimum for G_s_α open states spans a larger range of distances between G_s_α α5 and β_2_AR, indicating a larger chance for dissociation. Moreover, we found fewer interacting amino acid residues between α5 and β_2_AR and a bent α5 conformation in run 2 with an open state compared with run 1 where G_s_α is mostly in a closed state. Also, the number of interacting amino acid residues at the G_s_–β_2_AR binding interface shows a clear trend of decrease in the longer run, run 3, also possibly suggesting a partial G_s_ dissociation (*SI Appendix*, Fig. S13). Altogether, we found that the opening of G_s_α favors its partial dissociation from β_2_AR but is not sufficient. The interdomain rearrangement, namely, the stacking of G_s_α helices α1 and α5, is necessary for the partial G_s_ dissociation process. We have to mention that we only considered nucleotide-free and receptor-bound open-in G_s_ initial state in this work. The effect of GTP/GDP binding to the G_s_ conformational transitions and dissociation will be evaluated in a follow-up study.

**Table 2.** MM–PBSA interaction free energies between β_2_AR and G_s_ (in kcal/mol) along with their SEM computed using block averages, enthalpic (Δ*H*) and entropic (−*T*Δ*S*) components (based on the last 2 μs of Anton trajectories)

System	Time	Δ*H*	−*T*Δ*S*	Δ*G* ± SEM
β_2_AR–G_s_ – run1	3.0–5.0 μs	−145.4	105.1	−40.3 ± 8.2
β_2_AR–G_s_ – run2	3.0–5.0 μs	−111.8	82.9	−28.9 ± 8.6
β_2_AR–G_s_ – run3	5.5–7.5 μs	−154.6	105.4	−49.2 ± 17.2
β_2_AR–G_s_ – run4	3.0–5.0 μs	−109.6	83.6	−26.0 ± 4.9

## Conclusions

Combining all-atom multi-microsecond–long MD simulations with a posteriori implicit-solvent MM–PBSA calculations, we found that G_s_ binding to β_2_AR can stabilize the NE(+) binding to β_2_AR through stabilizing the structure of the active β_2_AR conformation. Different binding poses and partial dissociation of NE(+) were captured in both free and G_s_-bound β_2_AR systems. The partial dissociation of NE(+) can be attributed to the altered β_2_AR structure due to its interactions with G_s_, evidenced by the variances of β_2_AR RMSD values. The waggling of NE(+) binding to β_2_AR, i.e., presence of alternative binding poses closer to extra- or intracellular sides than the orthosteric binding site, was found to be related to the G_s_α conformational transition to a semi-closed or closed state. Using all-atom MD simulations, we also observed interaction between β_2_AR's ICL3 and G_s_ which caused the partial unwinding of the G_s_α α5 helix in the open-in state of this subunit, suggesting the important role of ICL3 in the G_s_ dissociation. ICL3 was included in our models but usually missing in the available PDB structures (7, 8, 53); thus, very limited information can be found about its function in related works (6, 12, 41). We also captured multiple closed and semi-closed conformations of the G_s_α subunit in the β_2_AR–G_s_ system. These conformations are absent in previous simulation works (6, 23, 40, 41) and hard to obtain from experiments due to the highly dynamic nature of G_s_αAH (8, 56). Our simulation data indicate the possibility of G_s_ closing before its partial dissociation from β_2_AR, which was not observed in previous simulation studies to the best of our knowledge. However, the closed G_s_α conformation is less favorable compared with the open one in promoting the dislocation of G_s_α α5 from its β_2_AR binding site. Instead, the internal G_s_αRas domain stacking between helices α1 and α5 was found to be necessary. We found that the open G_s_α favors a more stacked α1 and α5 arrangement, which can drive the dissociation of G_s_α α5 from the receptor. Yet, the binding of guanine nucleotides may have a different effect on the G protein conformational changes and dislocation of G_s_α α5 from its receptor binding site, which will be evaluated in our subsequent studies. The results of this study may help explain molecular determinants and underlying mechanisms on why bound G_s_ protein can stabilize NE(+) binding to β_2_AR and how G protein dissociation from the receptor may commence in the nucleotide-free state. These questions are important for understanding the activation of GPCRs and their modulation by G protein interactions in normal physiological and pathophysiological conditions. Our results can also be used to inform the next generation of multiscale functional kinetic models of sympathetic nervous stimulation in cardiac myocytes and other excitable cells, which is a powerful tool to complement experimental and clinical research.

## Materials and Methods

### Protein Structures.

The 3D coordinates of adrenaline-bound β_2_AR were obtained from the published X-ray crystallographic structure (PDB: 4LDO) (59) to serve as a template for the activated receptor. The G_s_ heterotrimer template was obtained from the 3D coordinates of the crystal structure of β_2_AR–G_s_ complex (PDB: 3SN6) bound to agonist BI-167107 (P0G) (7). 3D coordinates were oriented via the Orientations of Proteins in Membranes (OPM) database (60). The adrenaline-bound receptor from PDB 4LDO was aligned to protein complex structure from PDB 3SN6 via UCSF Chimera (61) Matchmaker to replace the P0G-bound receptor of PDB 3SN6, then all ligands and nonphysiological proteins were removed. The resulting template, which combined the receptor of 4LDO with the G_s_ heterotrimer of 3SN6, was then assessed for clashing van der Waals radii before proceeding.

As the β_2_AR structure was published without 3D coordinates for the intracellular loop 3 (ICL3), this region as well as omitted regions of the published G_s_ model in PDB 3SN6 were remodeled using the ROSETTA implementation of fragment-based cyclic coordinate descent (CCD) (62, 63). Target sequences for de novo modeling of both the human β_2_AR and the G_s_ heterotrimer were obtained via UniProt (64). Rosetta comparative modeling (RosettaCM) was used with the Rosetta Membrane Energy Function to generate 10,000 decoy models of sequence-complete β_2_AR–G_s_ complex (65–67). Rosetta clustering analysis was used to assess convergence of decoys into different microstates using their RMSDs with a cluster radius of 2.5 Å. The lowest-energy decoy of the most populated cluster was selected as a model for further refinement.1,000 energy-minimized decoys were then generated from the sequence-complete model using the Rosetta Fast Relax application in conjunction with the membrane energy function (68). Relaxation was permitted only to residues that were modeled de novo. The lowest energy structure was then selected for ligand docking and MD simulations.

### Ligand Docking.

RosettaLigand (69) was used for all docking simulations of NE(+) to β_2_AR and β_2_AR–G_s_. Ligand rotamers and parameters were generated by OpenEye Omega (70) and ROSETTA scripts. A box size of 5 Å was used for ligand transformations along with 7 Å ligand distance cutoff for side chain and backbone reorientations (with <0.3 Å C_α_ restraint). 50,000 structures were generated in each run with top 10% selected by total score, out of which 50 lowest-interfacial score structures were validated for their convergence with the crystalized adrenaline of the original template structure 4LDO. Subsequent simulations were conducted using the lowest-interfacial score structures.

### Molecular Dynamics Simulations.

MD simulation systems of ~222,000 or ~302,000 atoms were generated using CHARMM-GUI (71–73) and consisted of β_2_AR protein or β_2_AR–G_s_ protein complex in lipid bilayers soaked by a 0.15-M NaCl aqueous solution. The outer bilayer leaflet contained pure 1-Palmitoyl-2-oleoylphosphatidylcholine (POPC), whereas the inner leaflet had ~70% POPC and ~30% 1-Palmitoyl-2-oleoylphosphatidylserine (POPS) as in a previous MD simulation study (23). The same ionizable protein residue protonation states, posttranslational modifications (lipidations and disulfide bonds based on UniProt data), and C- and N-protein termini as in that study (23) were used as well. All-atom biomolecular CHARMM36m protein (74), C36 lipid (75), and general CHARMM (CGENFF) (76) force field and TIP3P water (77) were used. CGENFF program (78, 79) was used to generate cationic norepinephrine, NE(+), force field parameters by analogy, which were validated and had to be optimized for one dihedral angle using an established quantum mechanics (QM)-based protocol (76).

MD simulations were run in the *NPT* ensemble at 310 K and 1 atm pressure using tetragonal periodic boundary condition. The systems were equilibrated for 90 ns with gradually reducing protein restraints in the first 40 ns using Nanoscale Molecular Dynamics (NAMD) (80). MD equilibration runs were then followed by multi-microsecond–long production runs on the Anton 2 (81) supercomputer or using enhanced sampling Gaussian-accelerated MD (GaMD) (57) runs. The GaMD module implemented in the NAMD (82) was applied to perform GaMD simulations, which included a 10-ns short conventional MD (cMD) simulation (after the previous 90 ns MD equilibration), used to collect potential statistics for calculating the GaMD acceleration parameters, 50-ns GaMD equilibration after adding the boost potential, and finally three independent GaMD production runs with randomized initial atomic velocities for each system. All GaMD simulations were run at the “dual-boost” level by setting the reference energy to the lower bound. The upper limit of the boost potential SD, σ_0_, was set to 6.0 kcal/mol for both the dihedral and the total potential energy terms. Simulation analyses were performed using VMD (50) and lab-generated codes. The PyReweighting toolkit (83) was used to reweight the PMF profiles based on the distances and angles for GaMD trajectories to account for the effect of the boost potential on GaMD simulated distributions. A bin size of 0.5 Å was used for the interatomic distances and 5° for angles. The cutoff was set to 10 configurations in one bin for 2D PMF calculations. For the Anton simulations, PMF profiles did not need to be reweighted.

### MM–PBSA Binding Energies.

Free energy calculations for β_2_AR–NE(+) binding and β_2_AR–G_s_ binding were performed using the Molecular Mechanics–Poisson–Boltzmann Surface Area (MM–PBSA) approach with all-atom MD simulation trajectories by MMPBSA.py program in Amber Tools (84). The Chamber module of ParmEd program was used to convert CHARMM-style forcefields to Amber-style forcefields (85). Aqueous solution (ionic strength 150 mM) and lipid membrane were treated implicitly using dielectric constants (water ε_w _= 80, lipid bilayer ε_l_ = 2, and protein ε_p_ = 4). Solvent probe radius is set to 1.4 Å and the atomic radii were set according to the converted force field parameters. To obtain the enthalpy (Δ*H*) contributions of solvation and gas-phase free energies, the particle-particle particle-mesh (P3M) procedure was used (86). These calculations were performed with implicit membrane, where the electrostatic energy includes both reaction filed and Coulombic electrostatic energies. Entropy was calculated separately by the interaction entropy method (87). This method was shown to increase the entropy calculation efficiency and possibly improve the accuracy of MM–PBSA in estimating protein–protein interactions (88). To use the interaction entropy method, gas-phase interaction energies including Coulombic electrostatic and van der Waals components were computed. In order to get the gas-phase Coulombic energy separated from the reaction filed energy contribution, each system energy was recalculated by using dielectric boundary surface charges method in the implicit ionic solution. In this study, we focused on trends in relative binding free energies for the same or similar (β_2_AR and β_2_AR–G_s_) protein systems, which may justify the usage of a standard MM–PBSA approach (84) along with interaction entropy calculations (87). However, to obtain more accurate absolute and relative protein–protein binding free energy estimates, we may need to use recently developed MM–PBSA method with a screened electrostatic energy (88) in subsequent studies.

To reweight the MM–PBSA energies computed from GaMD simulations, we used the PyReweighting toolkit (83) to generate a corresponding PMF ( W ) value for each bin of the energy histogram generated from the simulation trajectories as described above for distance and angle PMFs. The probability for each bin can then be computed as $P_{\mathrm{bin}}=e^{-\beta W}$ , where β = 1/(*k*_B_*T*), *k*_B_ is Boltzmann constant and *T* is temperature. The average MM–PBSA energy in the GaMD boost-potential biased ensemble (notated with an asterisk, $\langle E^{*}\rangle$ ) is then converted to the canonical ensemble value $\langle E\rangle$ using probabilities, $P_{\mathrm{bin}}$ , and energies, $E_{\mathrm{bin}}^{*}$ , for each bin as $\langle E\rangle \;=\;\frac{\sum_{bin=1}^{N}P_{\mathrm{bin}}E_{\mathrm{bin}}^{*}}{\sum_{bin=1}^{N}P_{\mathrm{bin}}}$ . The bin width was kept as 0.5 kcal/mol. Similar reweighting approach can be in principle applied to interaction entropies using a cumulant expansion approach outlined in (89), but results for our systems were found to be noisy and unreliable (divergent) due to domination of higher-order terms.

### Binding Pose Clustering.

The clustering for the NE(+) binding poses was performed by TTClust program (90). The trajectories were first aligned to the first frame of β_2_AR (without intracellular loop 3). The RMSDs of NE(+) between all pairs of frames were calculated and stored into a matrix. This matrix was then used to calculate a linkage matrix by the hierarchical cluster linkage function of the SciPy package (91). Ward’s method within the SciPy module was used to minimize the variance within clusters and allows more demarcated clusters to be obtained (90). K-means clustering with the Elbow algorithm was used to find the optimal number of clusters (90).

### Pearson’s Correlation Coefficients.

The Pearson’s correlation coefficients (values of *r*) shown in *SI Appendix*, Table S4 were calculated among the data points in Fig. 4 *A* and *B* collected from the average values of the last 2 μs of each Anton run.

The time-lag correlation analysis was performed using MATLAB version 2022b. Calculations of the Pearson’s correlation coefficients (values of *r*) were performed using the built-in corrcoef function. The lag time defines a delay between two different MD simulation measurements, e.g., the distance between two protein residues as compared to the angle between two protein domains. A lag time of zero indicates that the distance and angle observations are compared from the same simulation time points, whereas a lag time of 50 ns, for example, indicates that distance observations for time *t* will be compared with angle observations from time (*t* + 50) for the duration of the simulation. The lag time was varied from zero to half of the MD simulation length (e.g., 2.5 µs for a 5-µs–long simulation).

## Supplementary Material

Appendix 01 (PDF)

## Data Availability

All final study data are included in the article and/or *SI Appendix* with key molecular dynamics simulation and analysis data files and scripts available to download from Dryad digital repository at https://doi.org/10.25338/B89H1T.
