# Supplementary material for: Elucidation of a dynamic interplay between a beta-2 adrenergic receptor, its agonist, and stimulatory G protein
Source: Proc Natl Acad Sci U S A. 2023 Feb 28;120(10):e2215916120. doi: 10.1073/pnas.2215916120 (PMC10013855; doi:10.1073/pnas.2215916120)
Supplement: Supplementary file 1 — Appendix 01 (PDF) [file pnas.2215916120.sapp.pdf]

PNAS

Supporting Information for

Elucidation of a dynamic interplay between a beta-2 adrenergic receptor, its agonist and stimulatory G protein

Yanxiao Han[a], John R. D. Dawson[a,b] , Kevin R. DeMarco[a] , Kyle C. Rouen[a,b] , Slava Bekker[a,c] , Vladimir Yarov-Yarovoy[a,d] , Colleen E. Clancy[a,e] , Yang K. Xiang[e,f] , and Igor Vorobyov[a,e,1]

[a]Department of Physiology and Membrane Biology, University of California, Davis, CA 95616;

[b]Biophysics Graduate Group, University of California, Davis, CA 95616;

[c]Department of Science and Engineering, American River College, Sacramento, CA 95841;

[d]Department of Anesthesiology and Pain Medicine, University of California, Davis, CA 95616;

[e]Department of Pharmacology, University of California, Davis, CA 95616;

[f]VA Northern California Health Care System, Mather, CA 95655.

[1]To whom correspondence may be addressed. Email: ivorobyov@ucdavis.edu.

This PDF file includes:

Tables S1 to S5

Figures S1 to S14

Table S1. Molecular systems simulated, their simulation times (in ns or μs), average boost potentials (Δ*V*) and standard deviations (std) in kcal/mol for GaMD runs. All simulation systems were first subject to 90 ns long equilibration (eq) MD runs after which microsecond-long unbiased Anton 2 MD or enhanced sampling GaMD simulations commenced. See main text "*Materials and Methods*" section for more details.

<table>
<tr><th>System name</th><th>Eq MD</th><th colspan="2">Anton 2 MD</th><th>GaMD</th></tr>
<tr><td>β_2AR – NE(+)</td><td>90 ns</td><td colspan="2">2.5 μs</td><td>Run 1, 600 ns, ΔV = 14.56, std = 4.29
Run 2, 600 ns, ΔV = 15.14, std = 4.35
Run 3, 600 ns, ΔV = 14.65, std = 4.29</td></tr>
<tr><td rowspan="4">β_2AR – G_s – NE(+)</td><td rowspan="4">90 ns</td><td>Run 1</td><td>5.0 μs</td><td rowspan="4">Run 1, 600 ns, ΔV = 18.60, std = 4.78
Run 2, 600 ns, ΔV = 18.95, std = 4.86
Run 3, 600 ns, ΔV = 16.73, std = 4.64</td></tr>
<tr><td>Run 2</td><td>5.0 μs</td></tr>
<tr><td>Run 3</td><td>7.5 μs</td></tr>
<tr><td>Run 4</td><td>5.0 μs</td></tr>
</table>

Table S2. MM-PBSA interaction free energies (Δ*G*) between NE(+) and β_2AR or β_2AR – G_s (in kcal/mol) along with their standard errors of mean (SEM) computed using block averages, enthalpic (Δ*H*) and entropic (-*T*Δ*S*) components. Calculations were based on GaMD trajectories (600 ns each). See "*Materials and Methods*" section of the main text for a description of the reweighting procedure.

System	Δ*H*	-*T*Δ*S*	Δ*G* ± SEM	Reweighted Δ*H*
**β_2AR-GaMD-run1**	-26.00	8.93	-17.07±1.38	-25.96
**β_2AR-GaMD-run2**	-25.61	5.30	-20.31±0.44	-26.74
**β_2AR-GaMD-run3**	-26.25	7.71	-18.54±1.35	-27.69
**β_2AR-G_s-GaMD-run1**	-25.87	6.58	-19.29±1.46	-27.72
**β_2AR-G_s-GaMD-run2**	-24.22	7.15	-17.07±0.66	-22.21
**β_2AR-G_s-GaMD-run3**	-22.22	5.19	-17.03±0.54	-21.29

Table S3. Amino acid residue (AA) contact information between different components of G_s and β_2AR proteins from Anton 2 MD runs of β_2AR – G_s – NE(+) system. Close contacts are defined as AAs within 3 Å of each other. The stable contacts are defined as AA interacting more than 50% of the simulation time. The average percentage interaction time was calculated by averaging the interaction times of the stable AA contacts in the third column.

**Contacts**		**Number of stable contacts**	**Average percentage interaction time**
AA in β_2AR interact with $G_s\alpha$ α5	Run 1	26	86.7%
	Run 2	**22**	**85.0%**
	Run 3	25	89.5%
	Run 4	25	88.5%
AA in $G_s\alpha$ α5 interact with β_2AR ICL3	Run 1	4	53.0%
	Run 2	3	69.8%
	Run 3	4	67.2%
	Run 4	4	62.9%
AA in β_2AR ICL3 interact with $G_s\alpha$ α5	Run 1	3	65.7%
	Run 2	3	72.5%
	Run 3	3	66.6%
	Run 4	2	75.3%
AA in β_2AR ICL3 interact with G_s	Run 1	5	70.6%
	Run 2	9	88.1%
	Run 3	10	78.8%
	Run 4	3	72.7%

Table S4. Pearson correlation coefficients (*r*) calculated for any two MD simulation averaged geometric criteria characterized in main-text Figure 4 based on Anton 2 MD runs of β_2AR – G_s – NE(+) system: **A** – $G_s\alpha$ A161 to E299 distance, **B** – angle between two vectors of $G_s\alpha$AH and $G_s\alpha$Ras domains, **C** – $G_s\alpha$AH and $G_s\alpha$Ras interdomain distance, **D** – β_2AR NpxxY to $G_s\alpha$ α5 distance, **E** – β_2AR to $G_s\alpha$ α5 distance, **F** – $G_s\alpha$ α1 to α5 distance.

Row #	A and B				
1	0.61				
	A and C	B and C			
2	0.99	0.69			
	A and D	B and D	C and D		
3	-0.36	-0.71	-0.46		
	A and E	B and E	C and E	D and E	
4	0.53	0.07	0.46	0.55	
	A and F	B and F	C and F	D and F	E and F
5	-0.65	-0.63	-0.65	-0.06	-0.80

Table S5. MM-PBSA interaction free energies (ΔG) between β_2AR and G_s (in kcal/mol), along with their standard errors of mean (SEM) computed using block averages, enthalpic (ΔH) and entropic ($-T\Delta S$) components based on GaMD trajectories (600 ns each). See the "*Materials and Methods*" section of the main text for a description of the reweighting procedure.

System	ΔH	$-T\Delta S$	ΔG±SEM	Reweighted ΔH
**β_2AR-G_s–GaMD-run1**	-142.3	97.9	-44.4±11.9	-144.4
**β_2AR-G_s–GaMD-run2**	-154.2	98.8	-55.3±13.8	-135.0
**β_2AR-G_s–GaMD-run3**	-119.5	94.4	-25.1±18.3	-132.2

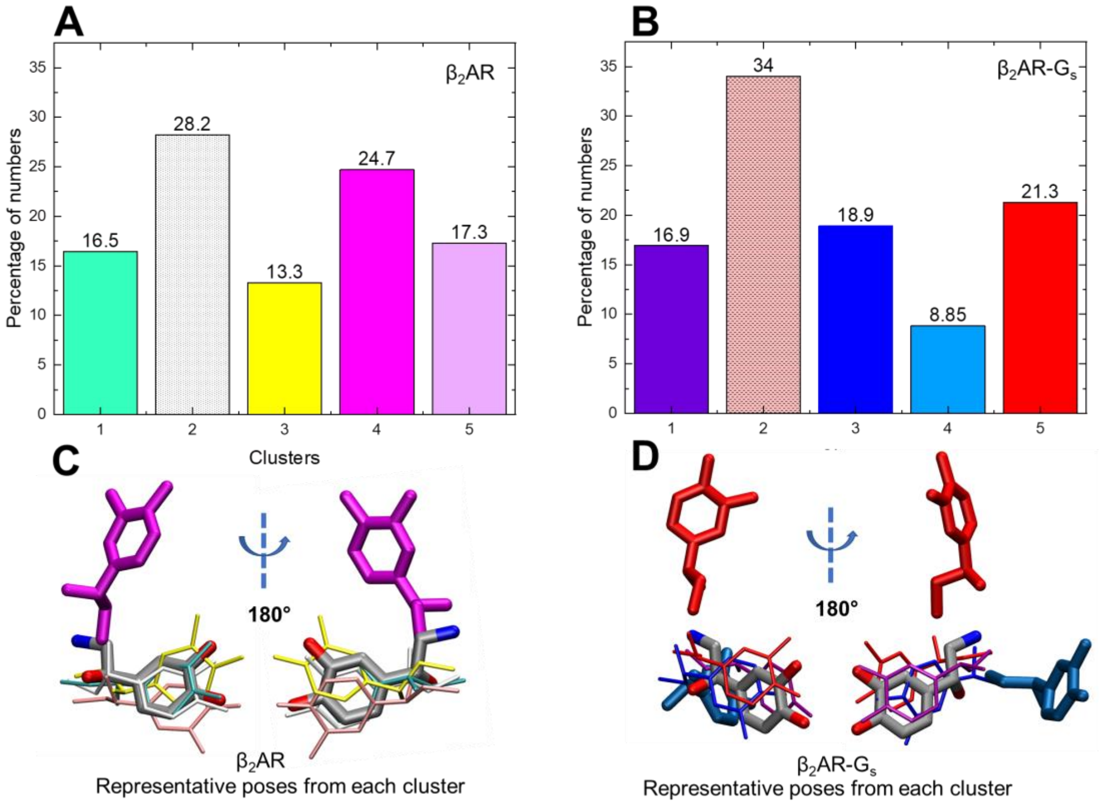

Fig. S1. (A) Clustering for binding poses of NE(+) in β$_2$AR Anton 2 run, percentage of pose numbers out of all poses in each cluster is shown on top of each bar. **(B)** Clustering for binding poses of NE(+) in β$_2$AR-G$_s$ (Four Anton 2 runs combined), percentage of pose numbers out of all poses in each cluster is shown on top of each bar. **(C)** Representative binding poses found for β$_2$AR, the coloring of molecules matches the histogram in (A), the white molecule corresponding to cluster 2 in (A). **(D)** Representative binding poses for β$_2$AR-G$_s$, the coloring of molecules matches the histogram in (B), the red molecule with thin bonds corresponds to cluster 2.

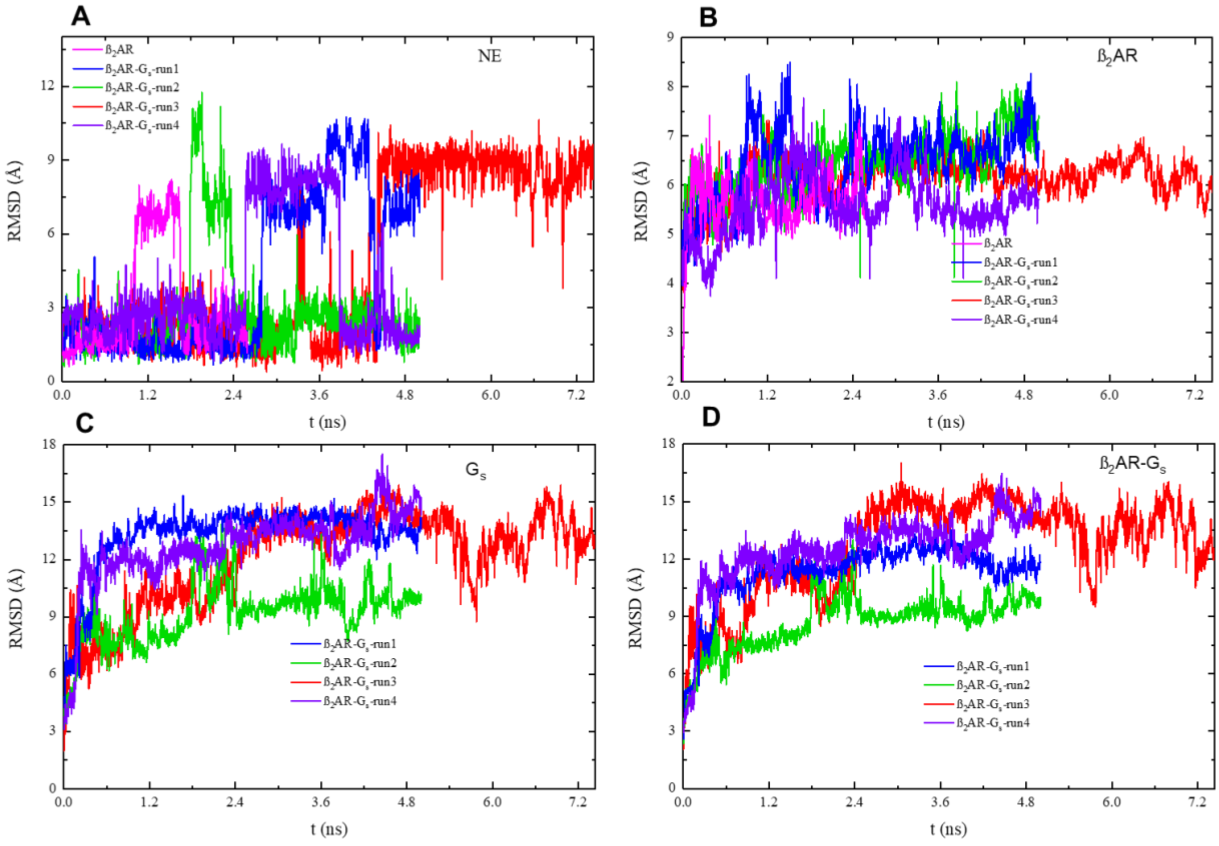

Fig. S2. RMSD time series of **(A**) NE(+) in different Anton 2 runs, trajectories were aligned to the β_2AR without loops with the first frame as reference; **(B**) β_2AR in different Anton 2 runs, trajectories were aligned to β_2AR with the first frame as reference; **(C)** G_s in different Anton 2 runs, trajectories were aligned to G_s with the first frame as reference; **(D)** β_2AR-G_s complex in different Anton 2 runs, trajectories were aligned to β_2AR-G_s with the first frame as reference.

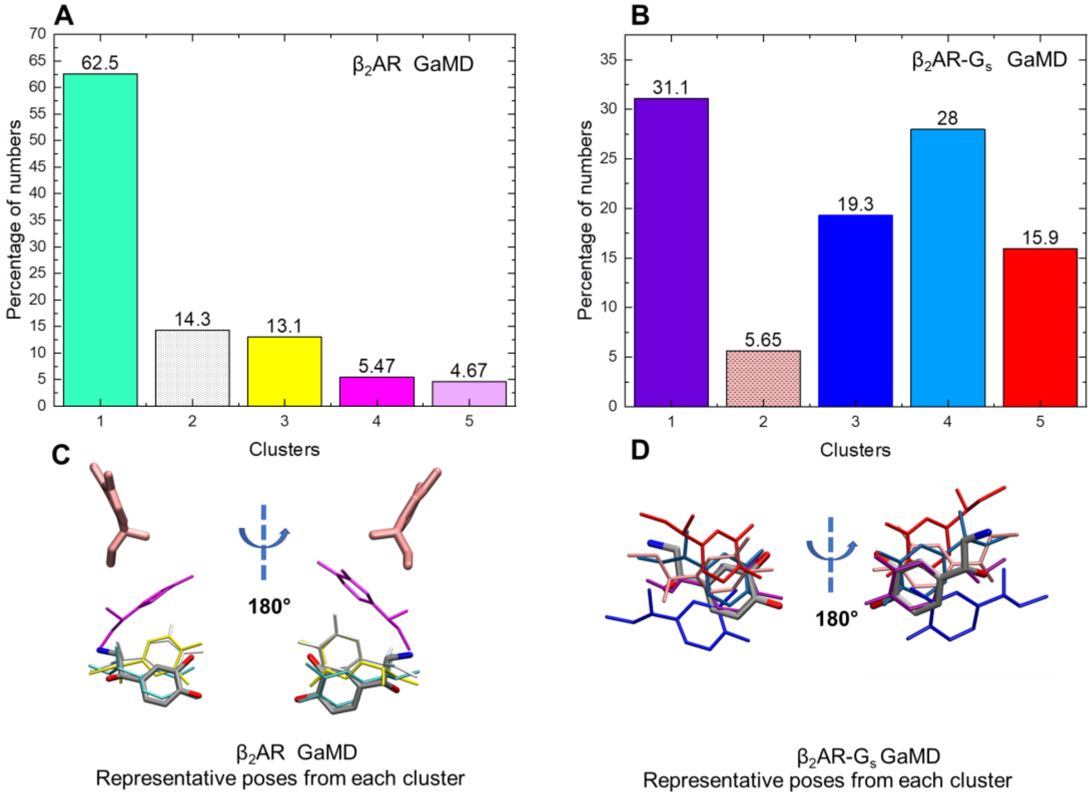

Fig. S3. (A) Clustering for binding poses of NE(+) in β_2AR GaMD runs, percentage of pose numbers out of all poses in each cluster is shown on top of each bar. **(B**) Clustering for binding poses of NE(+) in β_2AR-G_s GaMD runs, percentage of pose numbers out of all poses in each cluster is shown on top of each bar. **(C)** Representative binding poses found for β_2AR, the coloring of molecules matches the histogram in (A), the white molecule corresponds to cluster 2 in (A). **(D)** Representative binding poses for β_2AR-G_s, the coloring of molecules matches the histogram in (B), the pink molecule corresponds to cluster 2.

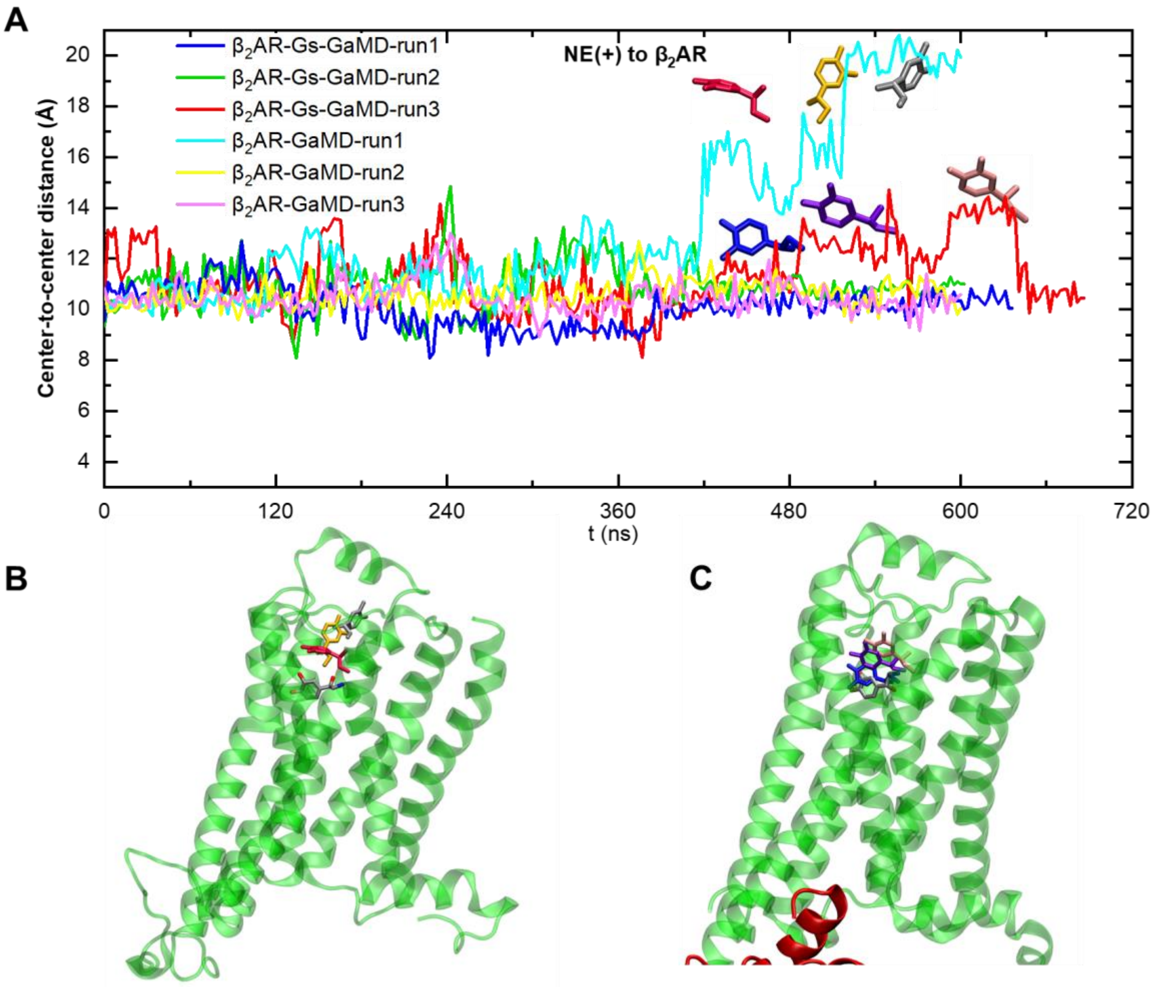

Fig. S4. **(A)** Time series of center-to-center distance between NE(+) and β_2AR geometric centers based on GaMD simulations; **(B)** Representative binding poses of NE(+) from β_2AR-GaMD-run1 (NE(+) colors correspond to those in panel A); **(C)** Representative binding poses of NE(+) from β_2AR-G_s-GaMD-run3 (NE(+) colors correspond to those in panel A).

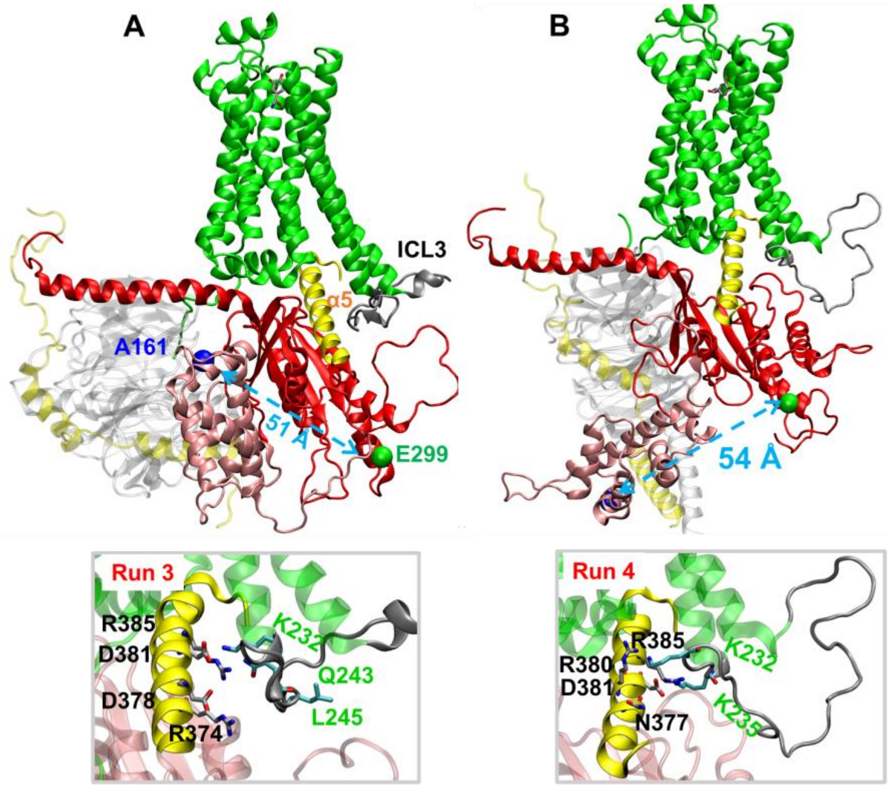

Fig. S5. All-atom Anton 2 MD simulations of the active state of the human β_2AR-G_s complex with NE(+) bound. **(A)** run 3 with the inset at the bottom. **(B)** run 4 with the inset at the bottom. Final structures from 5 μs long unbiased MD simulation runs on Anton 2. Individual protein chains / subunits are shown in the ribbon representation using different colors and labeled. $G_s\alpha$ α5 helix and β_2AR intracellular loop 3 (ICL3) are shown as yellow and dark gray. $G_s\alpha$ α-helical domain residue A161 and Ras-like domain residue E299 are shown as blue and green balls, and distances between them are shown by light-blue dashed arrows.

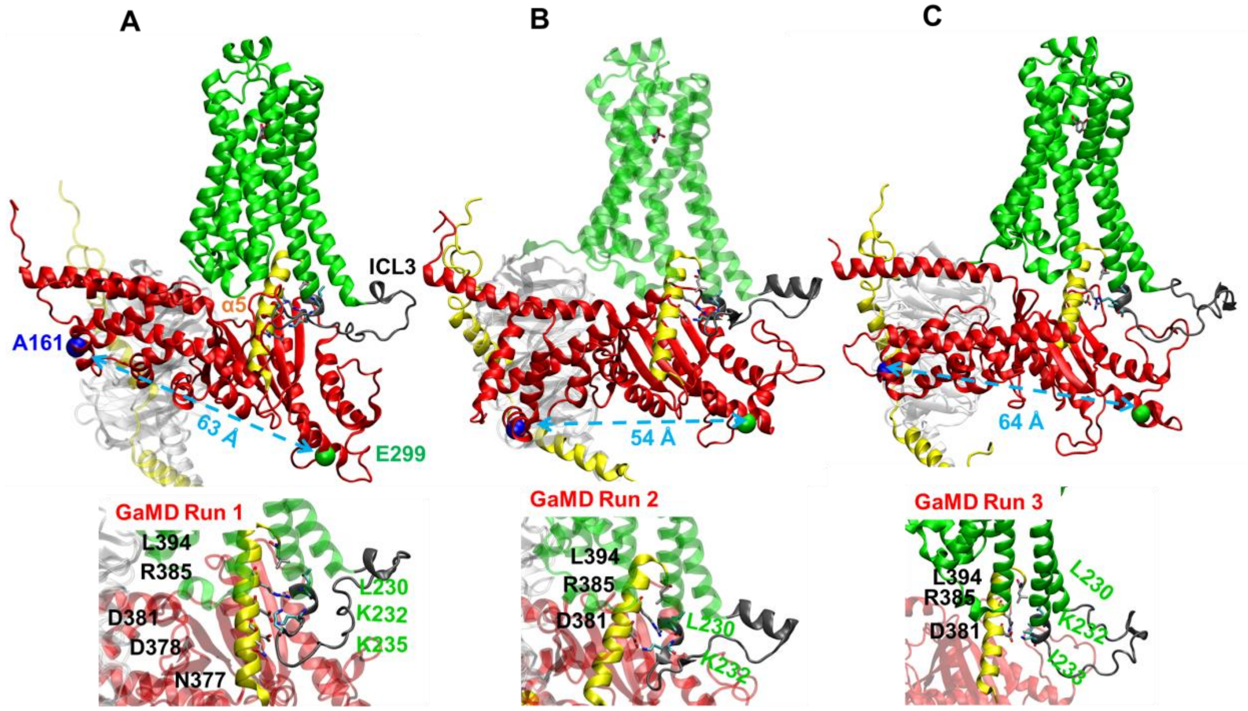

Fig. S6. All-atom GaMD simulations of the active state of the human β_2AR-G_s complex with NE(+) bound. **(A)** GaMD run 1 with the inset at the bottom. **(B)** GaMD run 2 with the inset at the bottom. **(C)** GaMD run 3 with the inset at the bottom. Final protein structures from 600-ns long GaMD simulation runs are shown. Individual protein chains / subunits are shown in the ribbon representation using different colors and labeled. $G_s\alpha$ α5 helix and β_2AR intracellular loop 3 (ICL3) are shown as yellow and dark gray. $G_s\alpha$ α-helical domain residue A161 and Ras-like domain residue E299 are shown in blue and green balls, and distances between them are shown by light-blue dashed arrows.

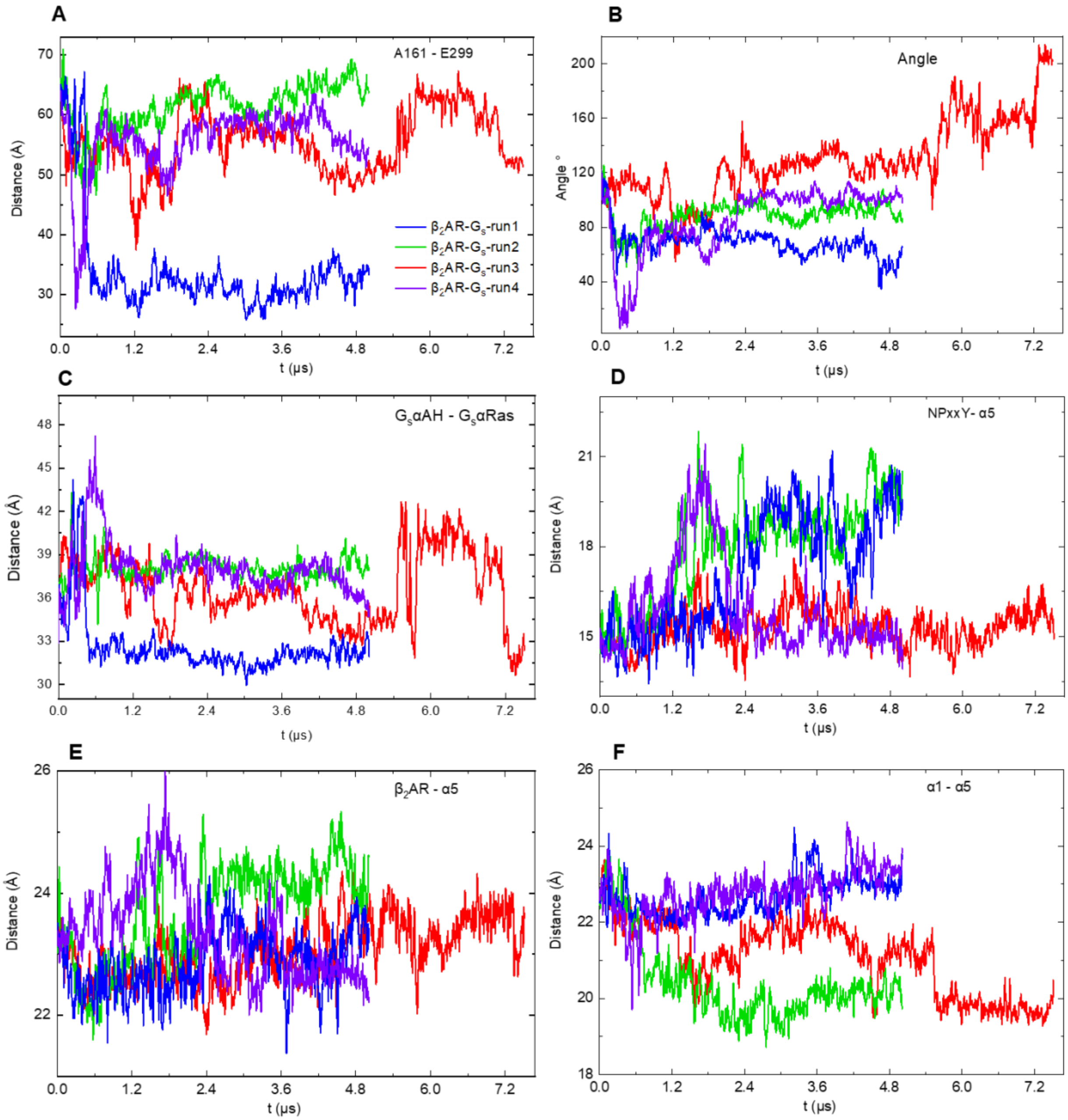

Fig. S7. Time series of geometric criteria from all-atom Anton 2 MD simulations of β_2AR-G_s-NE(+) system: **(A)** $G_s\alpha$ A161 to E299 distance indicating protein conformational changes (opening or closing); **(B)** angle between two vectors found in $G_s\alpha$AH and $G_s\alpha$Ras domains indicating the relative orientation of two domains. Vector 1 goes through $G_s\alpha$AH and A161 centers, vector 2 goes through $G_s\alpha$Ras and E299 centers (see main-text Figure 4C); **(C)** distance between $G_s\alpha$AH and $G_s\alpha$Ras domains; **(D)** distance between NPxxY (on the TM7 of β_2AR) and $G_s\alpha$ α5 helix indicating possible partial β_2AR-G_s dissociation; **(E)** distance between β_2AR and $G_s\alpha$ α5 indicating possible partial β_2AR-G_s dissociation; **(F)** $G_s\alpha$ α1 to α5 distance indicating relative movement of α1 and α5 helices. (The geometric centers were used for the distance and angle measurements.)

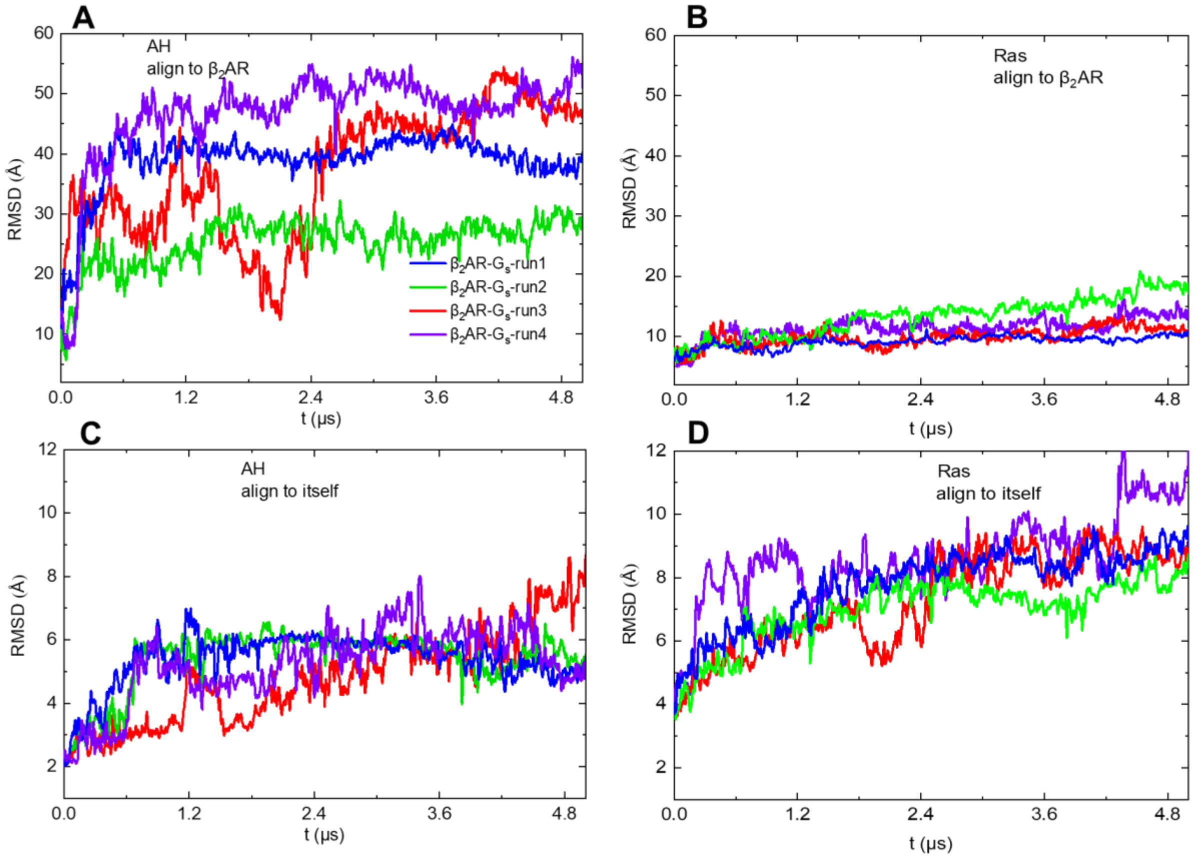

Fig. S8. RMSD time series from all-atom Anton 2 MD simulations of β_2AR-G_s-NE(+) system: **(A)** $G_s\alpha$AH domain C_α atoms aligned with respect to β_2AR; **(B)** $G_s\alpha$Ras domain C_α atoms aligned with respect to β_2AR; **(C)** $G_s\alpha$AH domain C_α atoms aligned with respect to its initial structure; **(D)** $G_s\alpha$Ras domain C_α atoms aligned with respect to its initial structure.

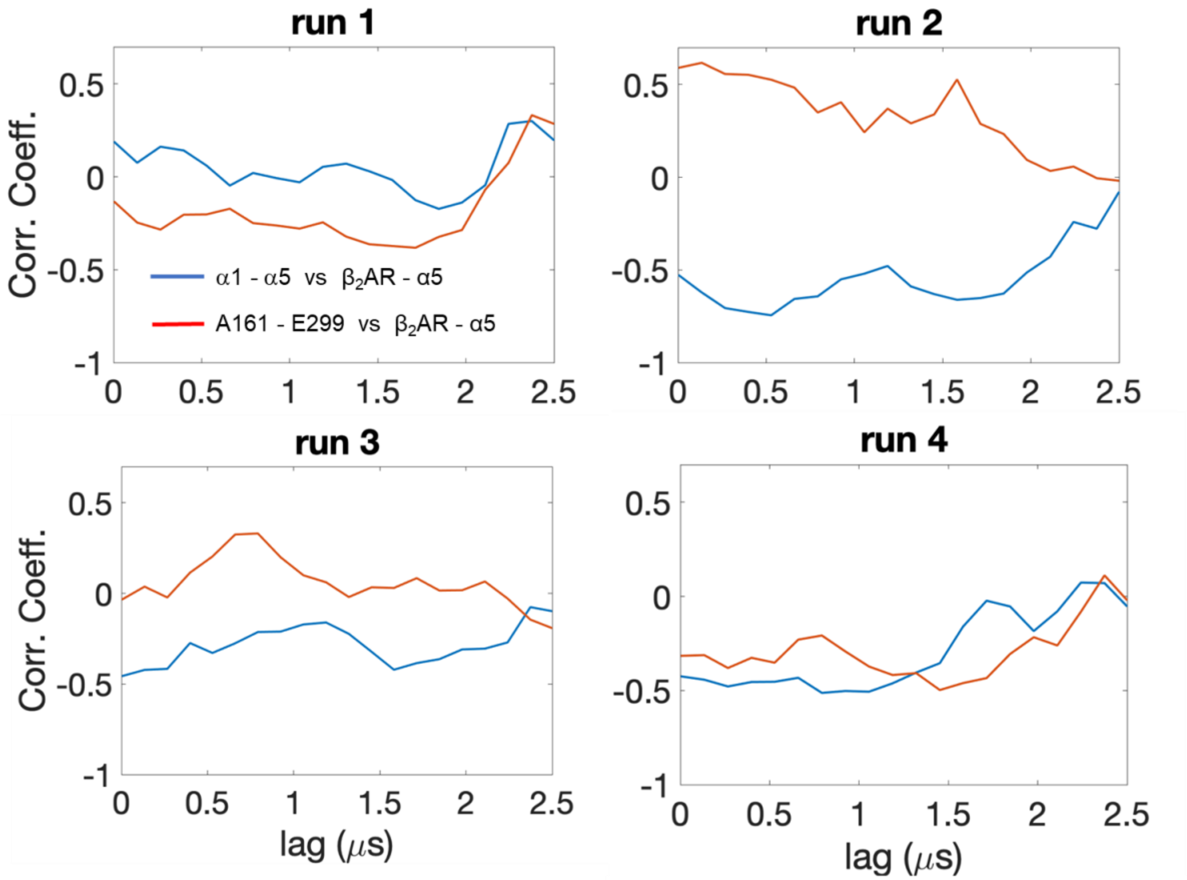

Fig. S9. Pearson correlation coefficients (Corr. Coeff) *r* as a function of lag time calculated for $G_s\alpha$ α1 - α5 distance vs. β_2AR - $G_s\alpha$ α5 distance (blue) and $G_s\alpha$ A161 - E299 distance vs. β_2AR - $G_s\alpha$ α5 distance (red). These data are based on all-atom Anton 2 MD simulations of β_2AR-G_s-NE(+) system.

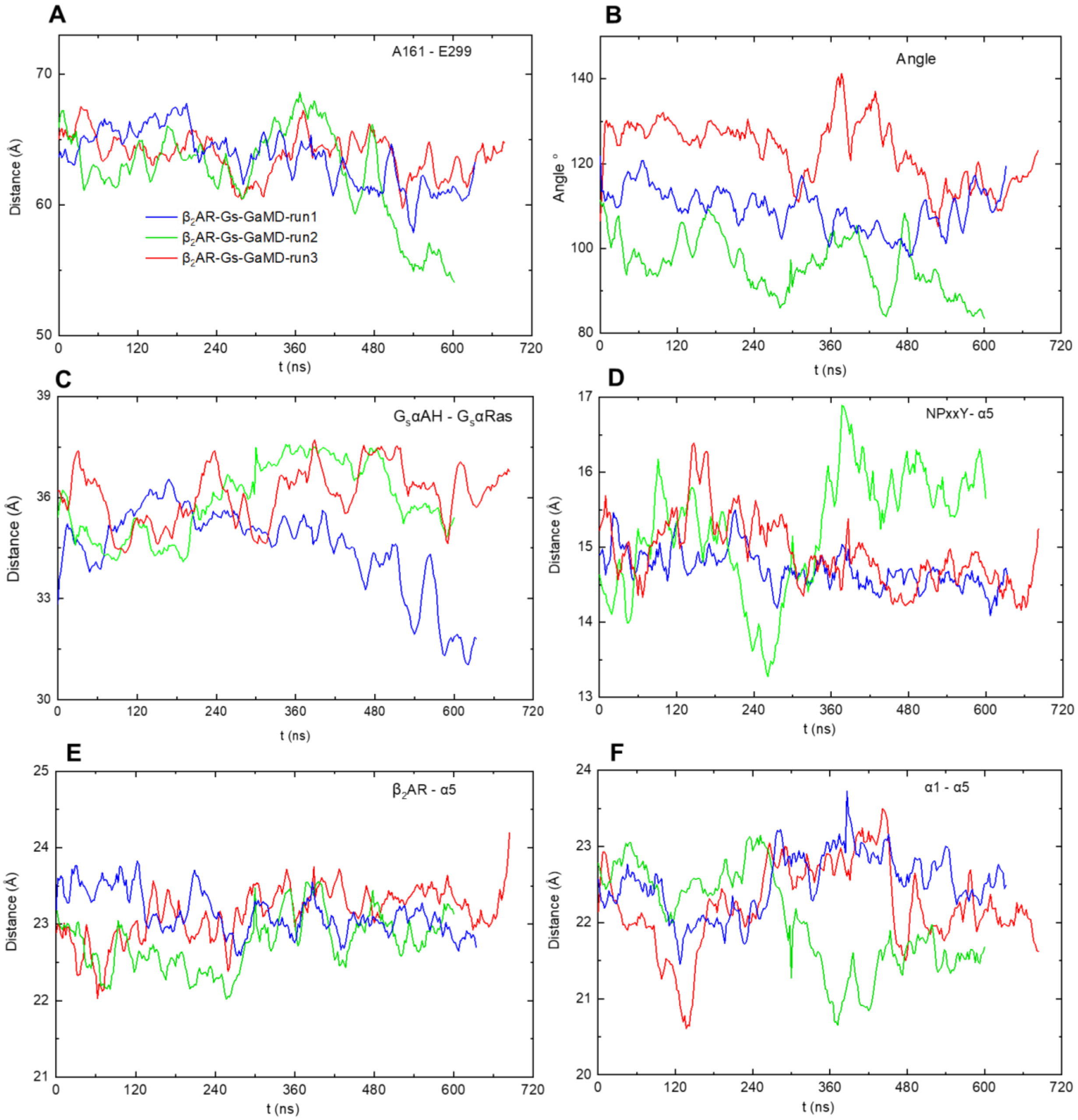

Fig. S10. Time series of geometric criteria from all-atom GaMD simulations of β_2AR-G_s-NE(+) system: **(A)** $G_s\alpha$ A161 to E299 distance indicating protein conformational changes (opening or closing); **(B)** Angle between two vectors found in $G_s\alpha$AH and $G_s\alpha$Ras domains indicating the relative orientation between the two domains. Vector 1 goes through $G_s\alpha$AH and A161 centers, vector 2 goes through $G_s\alpha$Ras and E299 centers (see main text Fig. 4C); **(C)** Distance between $G_s\alpha$AH and $G_s\alpha$Ras domains; **(D)** Distance between NPxxY (on the TM7 of β_2AR) and $G_s\alpha$ α5 indicating possible partial β_2AR-G_s dissociation; **(E)** Distance between β_2AR and $G_s\alpha$ α5 indicating possible partial β_2AR-G_s dissociation; **(F)** Distance between $G_s\alpha$ α1 and α5 indicating relative movement of helices α1 and α5. (The geometric centers were used for the distance and angle measurements.)

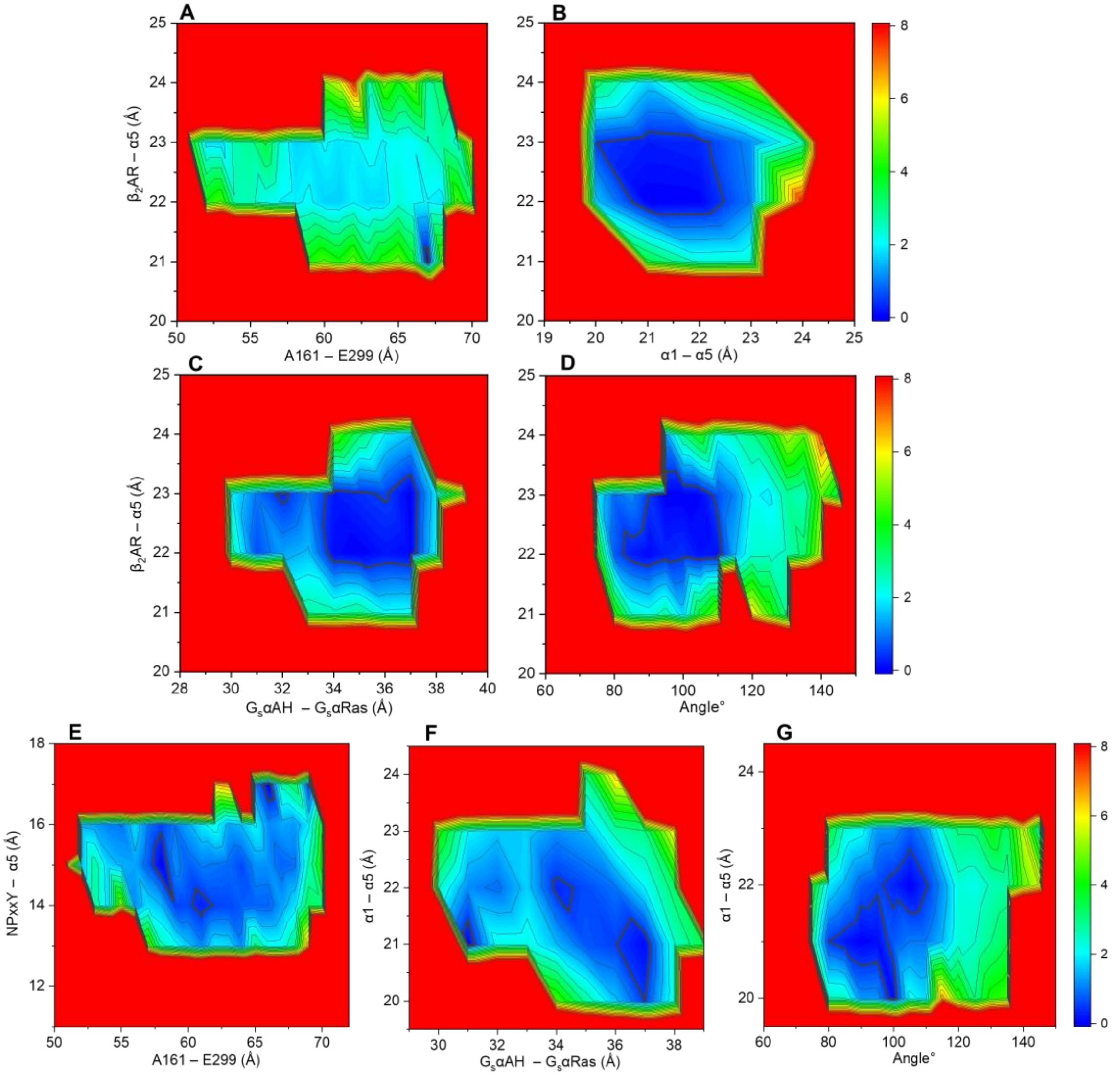

Fig. S11. 2D potential of mean force (PMF) or free energy profiles (in kcal/mol) based on Gsα conformation and its possible partial dissociation from β_2AR from all-atom GaMD simulations of the active state of the human β_2AR-G_s complexes with bound NE(+): **(A)** A161 to E299 distance indicating Gsα open or closed conformation is shown as *X*-axis. Distance between Gsα α5 and β_2AR indicating possible partial β_2AR-G_s dissociation is shown as *Y*-axis. **(B)** Gsα α1 to α5 distance is shown as *X*- axis. Distance between α5 and β_2AR indicating possible partial β_2AR-G_s dissociation is shown as *Y*-axis. **(C)** Distance between GsαAH and GsαRas is set as *X*-axis. Distance between Gsα α5 and β_2AR indicating possible partial β_2AR-G_s dissociation is shown as *Y*-axis. **(D)** Angle between two vectors, one from GsαAH and the other from GsαRas, is set as X-axis (shown in Figure 4C). Distance between α5 and β_2AR indicating possible partial β_2AR-G_s dissociation is shown as *Y*-axis. **(E)** Distance between Gsα A161 and E299 is shown as *X*-axis. β_2AR NPxxY to Gsα α5 distance is shown as *Y*-axis. **(F)** Distance between GsαAH and GsαRas is set as *X*-axis. Distance between Gsα α5 and α1 is shown as *Y*-axis. **(G)** Angle between two vectors, one from GsαAH and the other from GsαRas (shown in Figure 4C), is set as *X*-axis. Distance between G_s α5 and α1 is shown as *Y*-axis. All data are from GaMD simulations. (The geometric centers were used for the distance and angle measurements.)

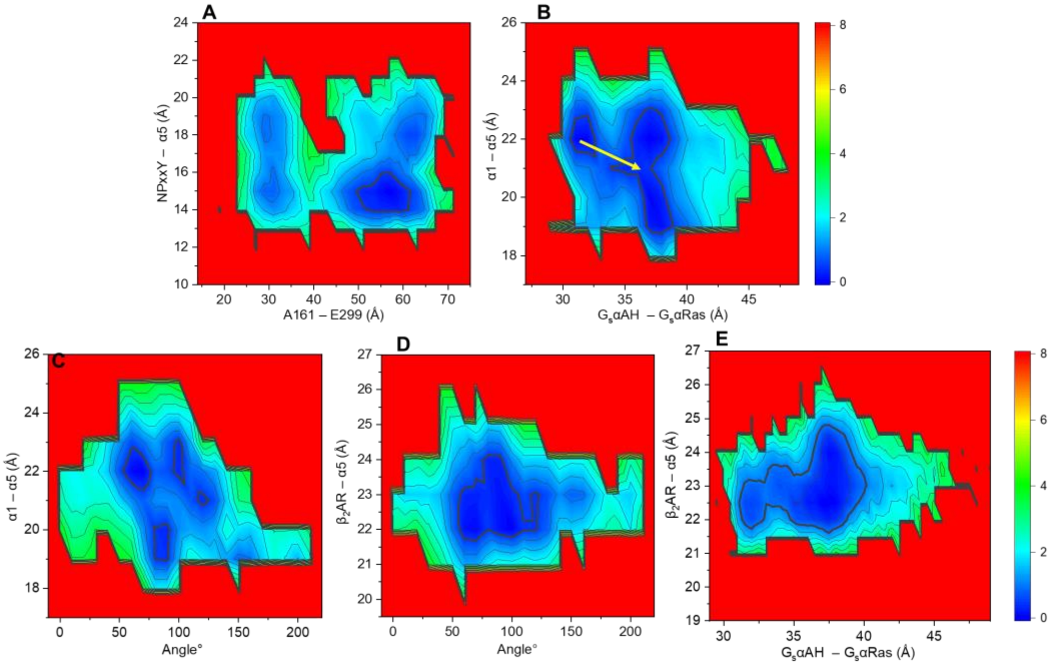

Fig. S12. 2D potential of mean force (PMF) or free energy profiles (in kcal/mol) from all-atom Anton 2 MD simulations of the active state of the human β_2AR-G_s complexes with bound NE(+) . **(A)** Distance between $G_s\alpha$ A161 and E299 is shown as *X*-axis. β_2AR NPxxY to $G_s\alpha$ α5 distance is shown as *Y*-axis. **(B)** Distance between $G_s\alpha$AH and $G_s\alpha$Ras is set as *X*-axis. Distance between $G_s\alpha$ α5 and α1 is shown as *Y*-axis. **(C)** Angle between two vectors, one from $G_s\alpha$AH and the other from $G_s\alpha$Ras, is set as *X*-axis. Distance between $G_s\alpha$ α5 helix and α1 helix is shown as *Y*-axis. **(D)** Angle between two vectors, one from $G_s\alpha$AH domain and the other from $G_s\alpha$Ras domain, is set as *X*-axis; distance between Gsα α5 and β_2AR is shown as *Y*-axis. **(E)** Distance between $G_s\alpha$AH and $G_s\alpha$Ras domains is set as *X*-axis; distance between α5 and β_2AR is shown as *Y*-axis. (The geometric centers were used for the distance and angle measurements.)

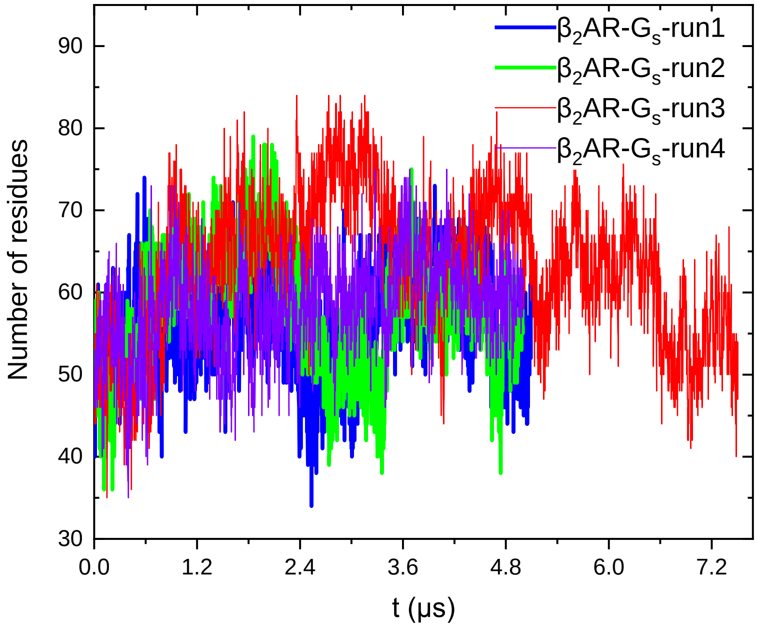

Fig. S13. Time series of the number of amino acid residues (AAs) in the binding interface between β_2AR and G_s from all-atom Anton 2 MD simulations of β_2AR-G_s-NE(+) system. The AAs in the binding interface were defined as those within 3 Å of either β_2AR or G_s.

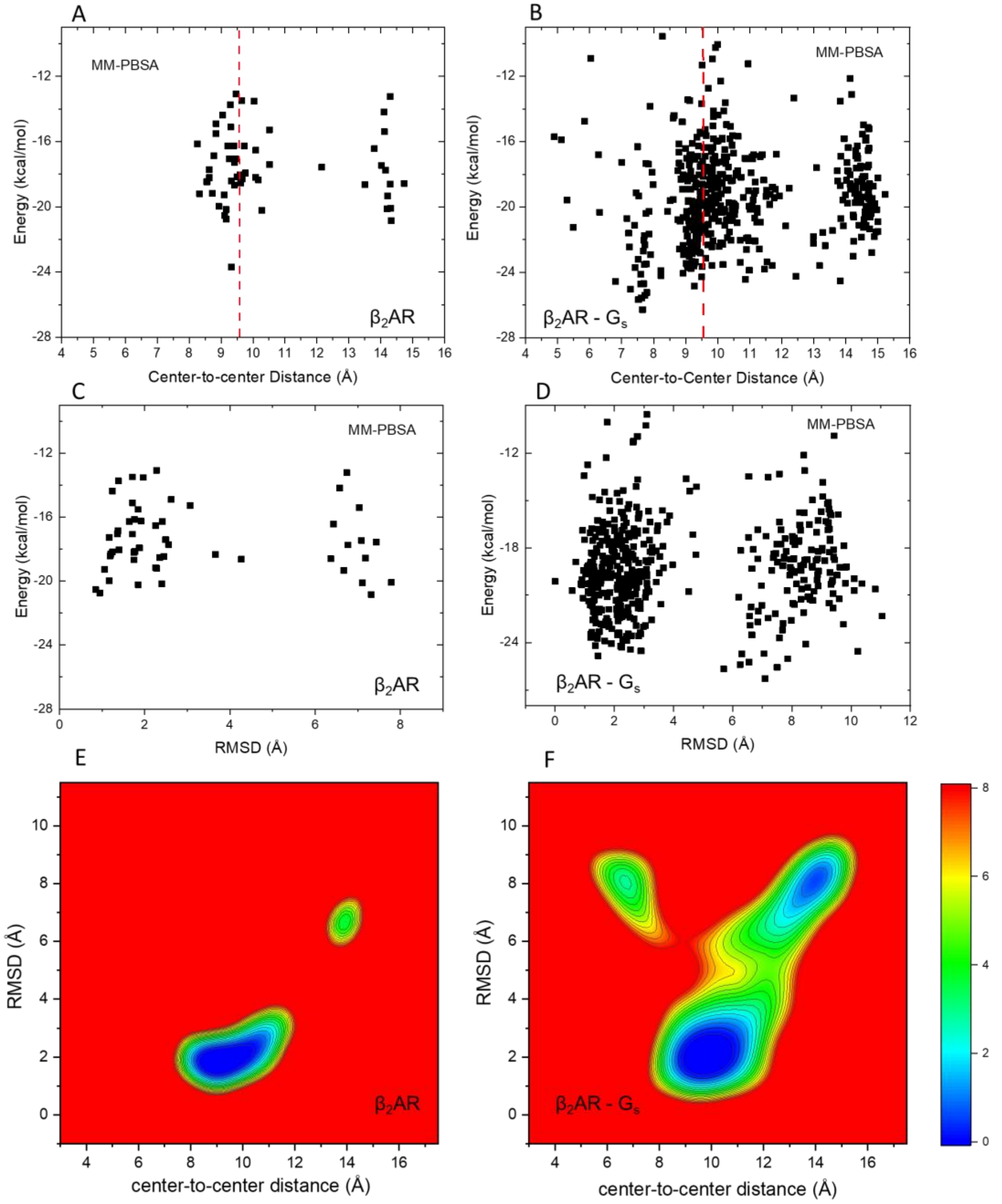

Fig. S14. **(A)** Scatter plot of MM-PBSA binding energies between NE(+) and β$_2$AR with their center-to-center distances in β$_2$AR only system. **(B)** Scatter plot of MM-PBSA binding energies between NE(+) and β$_2$AR with their center-to-center distances in β$_2$AR-G$_s$ system. **(C)** Scatter plot of MM-PBSA binding energies between NE(+) and β$_2$AR with RMSDs of NE(+) in β$_2$AR only system. **(D)** Scatter plot of MM-PBSA binding energies between NE(+) and β$_2$AR with RMSDs of NE(+) in β$_2$AR-G$_s$ system. **(E)** 2D PMF based on RMSD of NE(+) and center-to-center distance between NE(+) and β$_2$AR captured in the β$_2$AR only system. **(F)** 2D PMF based on RMSD of NE(+) and center-to-center distance between NE(+) and β$_2$AR captured in the β$_2$AR-G$_s$ systems. All plots are based on Anton 2 simulations, the vertical red dashed line in panels A and B indicates the initial center-to-center distance between NE(+) and β$_2$AR. (The geometric centers were used for the distance measurements.)
